# Model peptide for anti-sigma factor domain HHCC zinc fingers: high reactivity toward ^1^O_2_ leads to domain unfolding†
†Electronic supplementary information (ESI) available: Experimental details for peptide synthesis and characterization, absorption and circular dichroism analyses of Zn^2+^ binding, H_2_O_2_ oxidation, ^1^O_2_ oxidation and solution structure determination by NMR. See DOI: 10.1039/c9sc00341j


**DOI:** 10.1039/c9sc00341j

**Published:** 2019-02-21

**Authors:** Valentin Chabert, Vincent Lebrun, Colette Lebrun, Jean-Marc Latour, Olivier Sénèque

**Affiliations:** a Univ. Grenoble Alpes , CNRS , CEA , BIG , LCBM (UMR 5249) , F-38000 Grenoble , France . Email: olivier.seneque@cea.fr; b Univ. Grenoble Alpes , CEA , CNRS , INAC-SyMMES , F-38000 Grenoble , France

## Abstract

All organisms have to cope with the deleterious effects of reactive oxygen species. Some of them are able to mount a transcriptional response to various oxidative stresses, which involves sensor proteins capable of assessing the redox status of the cell or to detect reactive oxygen species. In this article, we describe the design, synthesis and characterization of Zn·L_ASD_(HHCC), a model for the Zn(Cys)_2_(His)_2_ zinc finger site of ChrR, a sensor protein involved in the bacterial defence against singlet oxygen that belongs to the family of zinc-binding anti-sigma factors possessing a characteristic H/C–X_24/25_–H–X_3_–C–X_2_–C motif. The 46-amino acid model peptide L_ASD_(HHCC) was synthetized by solid phase peptide synthesis and its Zn^2+^-binding properties were investigated using electronic absorption, circular dichroism and NMR. L_ASD_(HHCC) forms a 1 : 1 complex with Zn^2+^, namely Zn·L_ASD_(HHCC), that adopts a well-defined conformation with the Zn^2+^ ion capping a 3-helix core that reproduces almost perfectly the fold of the ChrR in the vicinity of its zinc site. H_2_O_2_ reacts with Zn·L_ASD_(HHCC) to yield a disulfide with a second order rate constant of 0.030 ± 0.002 M^–1^ s^–1^. Zn·L_ASD_(HHCC) reacts rapidly with singlet oxygen to yield sulfinates and sulfonates. A lower limit of the chemical reaction rate constant between Zn·L_ASD_(HHCC) and ^1^O_2_ was determined to be 3.9 × 10^6^ M^–1^ s^–1^. Therefore, the Zn(Cys)_2_(His)_2_ site of Zn·L_ASD_(HHCC) appears to be at least 5 times more reactive toward these two oxidants than that of a classical ββα zinc finger. Consequences for the activation mechanism of ChrR are discussed.

## Abstract 2

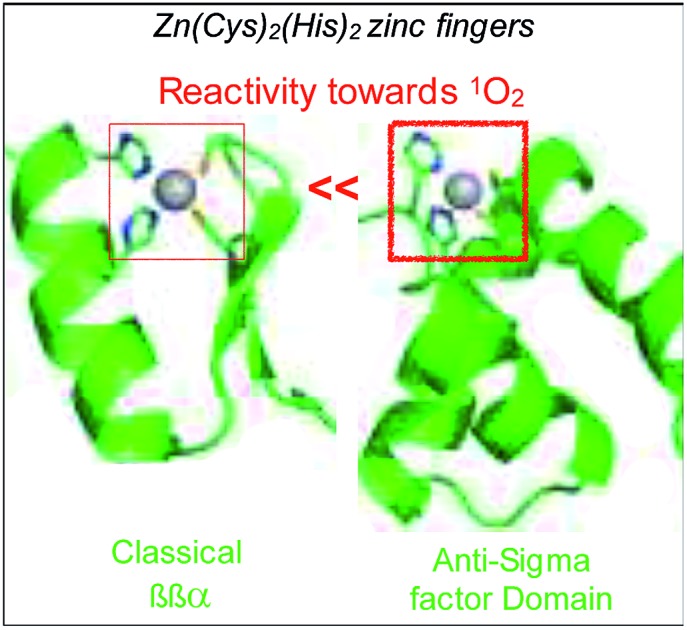
The Zn(Cys)_2_(His)_2_ site of the anti-sigma factor ChrR reacts rapidly with ^1^O_2_ supporting its involvement in ^1^O_2_ sensing by this protein.

## Introduction

**Fig. 1:**
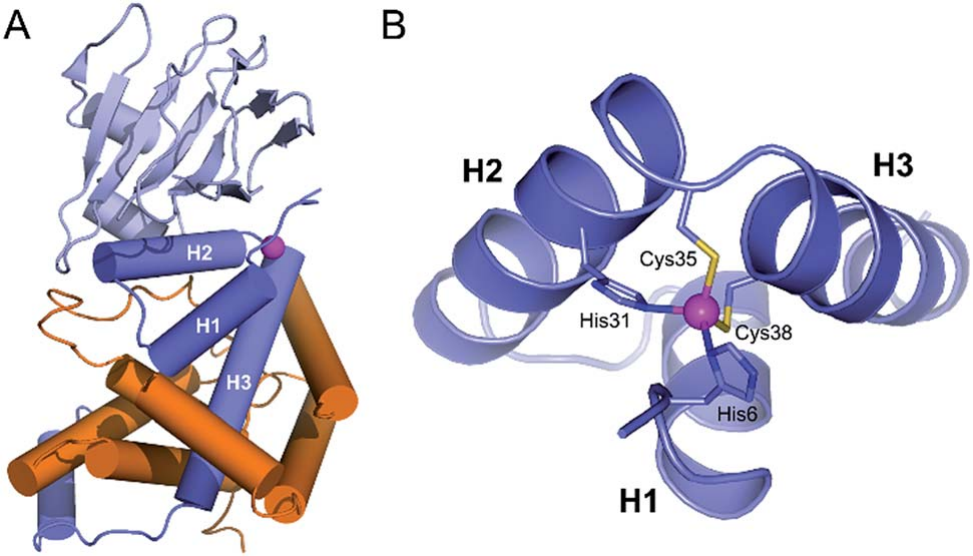
(A) Crystallographic structure of the ChrR–σ^E^ complex (pdb ; 2Z2S)^8^ showing the sigma factor σ^E^ in orange and the anti-sigma factor ChrR in blue with anti-sigma factor domain in dark blue and cupin-like domain in light blue. The zinc ion is shown in magenta. (B) View of the anti-sigma factor domain zinc finger site embedded within helices H1, H2 and H3, showing Zn^2+^-binding histidines and cysteines of the H–X_24_–H–X_3_–C–X_2_–C motif.

Organisms living in an aerobic environment have to cope with the deleterious effects of oxidative stress, which is caused by a family of molecules produced from dioxygen in the living cell, the so-called Reactive Oxygen Species (ROS).^1^ ROS are classified into two categories according to their production pathways: Type I for those arising from electron(s) addition to O_2_ (*e.g.*: superoxide anion O_2_˙^–^, hydrogen peroxide H_2_O_2_, hypochlorous acid HOCl, hydroxyl radical HO˙…) and Type II for electronically excited states of dioxygen arising from a physical activation of the ground (triplet) state (^3^O_2_).^2^–^4^ The lowest energy excited state of O_2_, commonly called singlet oxygen (^1^O_2_), is produced mainly in photosynthetic organisms. These organisms, as well as others like mammalians, have been shown to elicit a specific response to ^1^O_2_, demonstrating that sensing pathways exist for this ROS.^5^–^7^ One of the best characterized is that of *Rhodobacter sphaeroides*. It involves the protein σ^E^, a group IV sigma factor up-regulating the expression of the response against ^1^O_2_ stress.^8^–^11^ σ^E^ is negatively regulated by its cognate anti-sigma factor, ChrR. In normal conditions, the anti-sigma factor sequesters its cognate sigma factor (Fig. 1A). Under ^1^O_2_ stress, the anti-sigma factor undergoes a structural change and releases its sigma factor, which can then bind RNA polymerase, up-regulating the expression of specific genes involved in the response to ^1^O_2_ stress. The anti-sigma factor interacts with the sigma factor mainly *via* its N-terminal domain, named anti-sigma factor domain (ASD).^10^ It is estimated that 33% of the group IV sigma factors are regulated by an anti-sigma factor containing an anti-sigma factor domain. Among the predicted cytoplasmic group IV anti-sigma factors, 92% contain a conserved zinc binding motif H–X_3_–C–X_2_–C, classifying them in the zinc-binding anti-sigma factor (ZAS) subfamily.^8^

In 2011, Andreini *et al.* classified 93% of the 15 763 known structures of protein zinc sites into a minimum set of structural motifs.^12^ The two existing structures of ChrR^10^ could not enter in any of the families defined by Andreini, which demonstrates the singularity of the zinc finger site of their anti-sigma factor domain. ChrR contains an anti-sigma factor domain featuring a HHCC zinc finger site with a H–X_24_–H–X_3_–C–X_2_–C motif (Fig. 1B). Interestingly, RslA and RsrA, two ChrR homologs featuring also a zinc finger site – HHCC within a H–X_24_–H–X_3_–C–X_2_–C motif for RslA^13^ and CHCC within a C–X_25_–H–X_3_–C–X_2_–C motif for RsrA^14^ – are sensitive to oxidative conditions and trigger a response against oxidative stress.^13^–^16^ Noteworthy, the zinc site of RsrA is described as a redox switch, acting as a detection module for disulfide stress. A similar role was proposed for the zinc site of RslA to sense oxidative stress.^13^–^16^ It has been proposed that the oxidation of the ZAS protein zinc finger induces Zn^2+^ release and unfolding of the anti-sigma factor domain, leading to dissociation of the ZAS protein from its cognate sigma factor. However, it was recently demonstrated that oxidation of zinc-binding cysteine of the ZAS zinc finger is not always sufficient to dissociate the anti-sigma factor–sigma factor complex.^17^ Similarly, it has been hypothesized that the zinc site of ChrR could sense ^1^O_2_*via* zinc-bound thiol oxidation inducing dissociation of the anti-sigma factor domain from σ^E^, which is supported by the demonstration that (i) formation of the zinc finger site in the N-ter domain anti-sigma factor domain of ChrR is essential for the ChrR–σ^E^ complex formation, and (ii) the Zn-loaded anti-sigma factor domain alone is sufficient to sequester σ^E^. Additionally, it was also shown that the C-terminal cupin-like domain of ChrR is required for ^1^O_2_ response *in vivo*,^10^,^11^ but there is no data ruling out the involvement of the zinc finger site – and its oxidation – in these studies. The full mechanism of activation of ChrR by ^1^O_2_ remains to be elucidated.

We have previously shown that small metallopeptides modelling zinc finger sites are useful tools to gain interesting insights into the reactivity of zinc fingers toward ROS at the molecular level (oxidation products, kinetic rates).^18^–^20^ In particular, we have shown that a Zn(Cys)_4_ zinc finger of the treble clef family of zinc fingers could be efficiently oxidized by ^1^O_2_ 21 whereas ^1^O_2_ oxidation of the Zn(Cys)_2_(His)_2_ site of classical ββα zinc fingers is far less efficient.^22^ This questions the role of the Zn(Cys)_2_(His)_2_ zinc finger in ChrR. To address this question, we developed a 46-amino acid peptide modelling the anti-sigma factor domain zinc finger site of ChrR and we studied its reactivity towards H_2_O_2_ and ^1^O_2_. We show that its oxidation leads to Zn^2+^ release and peptide unfolding. Remarkably, the cysteines of this uncommon Zn(Cys)_2_(His)_2_ site found in ZAS are rapidly oxidized by ^1^O_2_ compared to classical ββα zinc fingers, in agreement with their putative involvement in ^1^O_2_ detection.

## Results

### Peptide design

**Fig. 2:**
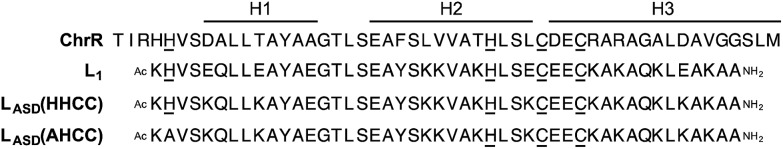
Sequence alignment of the N-terminal domain of ChrR (top) and the model peptides presented in this article, Zn^2+^-binding amino acids underlined.

To date, two crystal structures of anti-sigma factor domain-containing proteins displaying a zinc finger site (HHCC type) have been elucidated: ChrR^10^ and RslA.^13^ Anti-sigma factor domains display four α-helices and among them, three are involved in the constitution of the zinc finger site, which is displayed in Fig. 1B. These three helices (H1, H2 and H3) are held together by the chelation of a Zn^2+^ ion and by closely packed hydrophobic side chains within the heart of the 3-helix core. Helices H2 and H3 (Fig. 1B) form a knuckle bearing the H–X_3_–C–X_2_–C conserved motif, coordinating the Zn^2+^ ion. The fourth zinc ligand is a histidine residue located at the N-terminus resulting in an overall H–X_24_–H–X_3_–C–X_2_–C Zn^2+^-binding motif (Fig. 2). In order to reproduce the reactivity of a given zinc finger site, it is important to perfectly reproduce the various features of the site that can influence its reactivity. This includes the coordination sphere of the Zn^2+^ ion, the folding of the peptide around and the hydrogen bonds that are established with the Zn^2+^-bound sulfurs.^18^,^20^,^23^ It has been shown with RsrA that the anti-sigma factor domain can adopt different folds depending whether it is bound to its sigma-factor or not.^17^ Since our aim is to better understand if the reactivity of ChrR's zinc site could play a role in the sensing of ^1^O_2_, we aim here at reproducing the fold as complexed with σ^E^. Hydrophobic cores proximal to the zinc finger site can be important to enhance Zn^2+^ affinity to the model peptide and to ensure its proper folding.^24^ For these reasons, we decided to include the three helices in our model.

The design of the model peptides was based on the sequence and X-ray crystallographic structure of ChrR anti-sigma factor domain (Fig. 2),^10^ which was re-engineered as follows. (i) In the HH motif present at the N-terminus, only the second residue is involved in Zn^2+^ coordination as part of the canonical H–X_24_–H–X_3_–C–X_2_–C motif. In order to avoid scrambling of Zn^2+^ ligand, the first histidine of the HH motif was changed for a lysine. (ii) Helices H1, H2 and H3 present several hydrophobic side chains that point toward the exterior of the structure because they are involved in the interaction with the sigma factor σ^E^. In order to favour the correct fold of our model peptide and to ensure solubility, these hydrophobic amino acids were randomly changed for polar amino acids (E, Q or K). (iii) As β-branched amino acids such as threonine or valine may destabilize α-helices,^25^ two threonines located in helices H1 and H2, and pointing toward the exterior of the structure were changed for lysines. Similarly, glycines in helix H3, which may destabilize a helical fold, were changed for glutamine and alanines. (iv) The C-terminus of the peptide was amidated to remove the carboxylate charge that could destabilize the C-terminus of helix H3.^26^ (v) Non-essential aspartates and arginines were changed for glutamates and lysines to avoid side reactions during solid phase peptide synthesis (SPPS) and maximize synthesis yields. This led to peptide L_1_, whose sequence is displayed in Fig. 2. As a first try to model the anti-sigma factor domain zinc finger, this 46-amino acid peptide was synthetized by SPPS. Unfortunately, when dissolved in various buffers at pH around 7, L_1_ precipitated when Zn^2+^ was added. Mapping charged amino acids of the L_1_ sequence onto the 3-helix motif of ChrR using Pymol^27^ revealed that most of the negatively charged glutamate residues are clustered on one face of the structure whereas positively charged lysines are clustered on another face. Suspecting that this could be the reason for precipitation of the Zn^2+^ complex, the charged amino acids were re-distributed in the sequence for random disposition onto the 3-helix surface. This led to peptide L_ASD_(HHCC) (Fig. 2), which was synthetized by SPPS. Note that four pseudoproline dipeptides^28^ were used to avoid aggregation of the elongating chain during synthesis (see ESI† for details).

### Zinc binding and folding properties

**Fig. 3:**
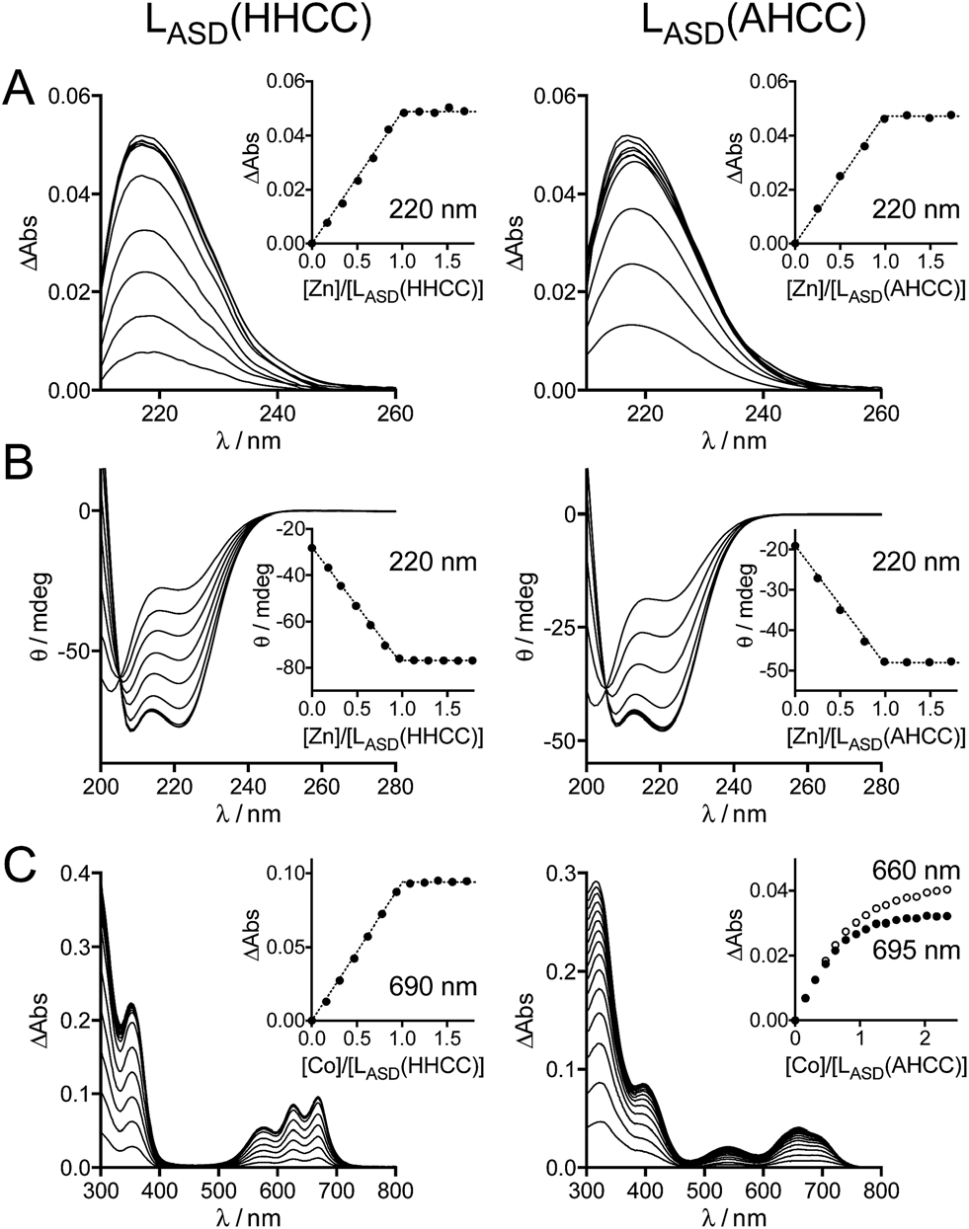
UV-Vis absorption and CD characterization of L_ASD_(HHCC) and L_ASD_(AHCC). (A) Absorption and (B) CD monitoring of Zn^2+^ titrations of L_ASD_(HHCC) (19 μM, left) and L_ASD_(AHCC) (14 μM, right) in phosphate buffer (20 mM, pH 7.0) containing TCEP (500 μM) with inserts showing the evolution of the absorbance at 220 nm. For absorption, the spectrum of the metal-free peptide was subtracted from each spectrum. (C) Absorption monitoring of Co^2+^ titrations of L_ASD_(HHCC) (153 μM, left) and L_ASD_(AHCC) (110 μM, right) in phosphate buffer (20 mM, pH 7.0) containing TCEP (500 μM) with inserts showing the evolution of the absorbance at 690 nm for L_ASD_(HHCC) and 660 and 695 nm for L_ASD_(AHCC).

The metal binding properties of L_ASD_(HHCC) were investigated by combination of UV-Vis absorption, circular dichroism (CD) and NMR. Titrations monitored by UV absorption were used to determine the stoichiometry of the peptide–metal complexes. Upon addition of Zn^2+^ in a buffered peptide solution, an increasing absorption band is observed at *ca.* 220 nm, which corresponds to Cys·S^–^ → Zn^2+^ ligand-to-metal charge transfer (LMCT) transitions (Fig. 3A).^24^ This signal increases linearly up to one equivalent of zinc and plateaus afterwards (inserts Fig. 3A). This indicates the formation of a 1 : 1 complex only, *i.e.* Zn·L_ASD_(HHCC). The intensity of this band is Δ*ε* = 6600 M^–1^ cm^–1^, which is in agreement with two zinc-bound cysteinates.^20^,^24^,^29^ To gain further insight into the coordination sphere of the Zn^2+^ ion, L_ASD_(HHCC) was titrated with Co^2+^, a Zn^2+^ ion surrogate commonly used to probe its geometry and coordination sphere. Absorption bands characteristic of Cys·S^–^ → Co^2+^ LMCT (in the range 220–400 nm) and d–d (in the range 500–700 nm) transitions appear upon coordination of Co^2+^ by L_ASD_(HHCC) (Fig. 3C). Similarly to the Zn^2+^ titration, only the 1 : 1 Co·L_ASD_(HHCC) complex is detected. The wavelengths (576, 627 and 670 nm) and intensity (*ε* = 620 M^–1^ cm^–1^ at 627 nm, *i.e. ε* > 300 M^–1^ cm^–1^) of the d*–*d transitions are in agreement with a tetrahedral Co(Cys)_2_(His)_2_ site.^20^,^24^,^30^,^31^ From these data, we can reasonably infer the formation of a tetrahedral 1 : 1 Zn·L_ASD_(HHCC) complex with (Cys)_2_(His)_2_ coordination. Thereafter, the folding of the peptide was investigated by CD (Fig. 3B). The CD spectrum of the metal-free L_ASD_(HHCC) shows an intense negative signal with minimum at *ca.* 200 nm and a shoulder at *ca.* 222 nm. This corresponds to a random coil peptide with a small content of helical fold. In agreement with absorption studies, upon titration of L_ASD_(HHCC) by Zn^2+^, evolution of the CD spectrum is observed up to 1 eq. Zn^2+^ with a clean isodichroic point at 205 nm, thereby confirming the formation of a complex with 1 : 1 stoichiometry only. The spectrum of Zn·L_ASD_(HHCC) is characteristic of a peptide with major helix content, as expected for this model. In order to determine the Zn^2+^ binding constant of L_ASD_(HHCC) at pH 7.0, *K*_ZnL_ASD_(HHCC)_ = [Zn·L_ASD_(HHCC)]/([Zn^2+^][L_ASD_(HHCC)]), CD titrations were performed in competition with EDTA and TPEN (1 eq.), two high-affinity Zn^2+^ chelators (*K*_ZnEDTA_ = 10^13.1^ M^–1^ and *K*_ZnTPEN_ = 10^14.9^ M^–1^ at pH 7.0). In the case of EDTA, *ca.* 95% of Zn^2+^ is bound to L_ASD_(HHCC) after addition of 1.0 eq. Zn^2+^*vs.* peptide (ESI†), indicating that L_ASD_(HHCC) binds Zn^2+^ tighter than EDTA but also that EDTA is not suitable for precise determination of the binding constant.^32^,^33^ In the case of TPEN, we noticed a slow precipitation of the Zn^2+^-free peptide in presence of this chelator, which precluded any competition experiment. Thus, only a lower estimate of the binding constant can be drawn from the EDTA competition, that is *K*_ZnL_ASD_(HHCC)_ ≥ 10^15.0^ M^–1^.

In order to confirm that the N-terminal histidine, which is remote from the core H–X_3_–C–X_2_–C binding motif in the sequence, is bound to the Zn^2+^ ion in our Zn·L_ASD_(HHCC) model, a peptide variant with the N-terminal histidine replaced by an alanine, namely L_ASD_(AHCC), was synthesized (Fig. 2 and ESI†). Upon Zn^2+^ titration, the formation of a 1 : 1 complex only is evidenced by both UV-Vis absorption and CD spectroscopies (Fig. 3A and B). The spectral features are very similar for both peptides, including LMCT absorption and CD spectra, either in their Zn-free and Zn-loaded forms. The intensity of the LMCT band (Δ*ε* = 8400 M^–1^ cm^–1^) is compatible with two zinc-bound cysteinates. Noteworthy, the CD spectrum of Zn·L_ASD_(AHCC) is very similar to that of Zn·L_ASD_(HHCC), indicating a similar helix content in the Zn-loaded form. Regarding Co^2+^ binding, analysis of the UV-Vis Co^2+^ titration of L_ASD_(AHCC) shows a completely different d–d transition pattern compared to L_ASD_(HHCC) with a two-step growing phase (the bands at 660 and 695 nm have the same intensity at the beginning of the titration (<0.5 eq.), then the band at 655 nm becomes the most intense, Fig. 3C), indicating the formation of both 2 : 1 and 1 : 1 Co/peptide species during the titration, with tetrahedral geometry as attested by the *ε* values above 300 M^–1^ cm^–1^ for the d*–*d transitions. Additionally, no plateau is observed after 1.0 eq., indicating a less stable 1 : 1 complex compared to Co·L_ASD_(HHCC). Finally, the binding affinity of L_ASD_(AHCC) for Zn^2+^ was assessed by competition with EDTA monitored by CD. When a 1 : 1 : 1 L_ASD_(AHCC)/EDTA/Zn^2+^ mixture is prepared, *ca.* 25% of Zn^2+^ is bound to L_ASD_(AHCC) *versus* 95% for L_ASD_(HHCC) in the same conditions (ESI†). This corresponds to a value of *ca.* 10^12^ for *K*_ZnL_ASD_(AHCC)_, indicating that the replacement of the N-terminal histidine by an alanine lowers the Zn^2+^ affinity by at least 3 orders of magnitude.

**Fig. 4:**
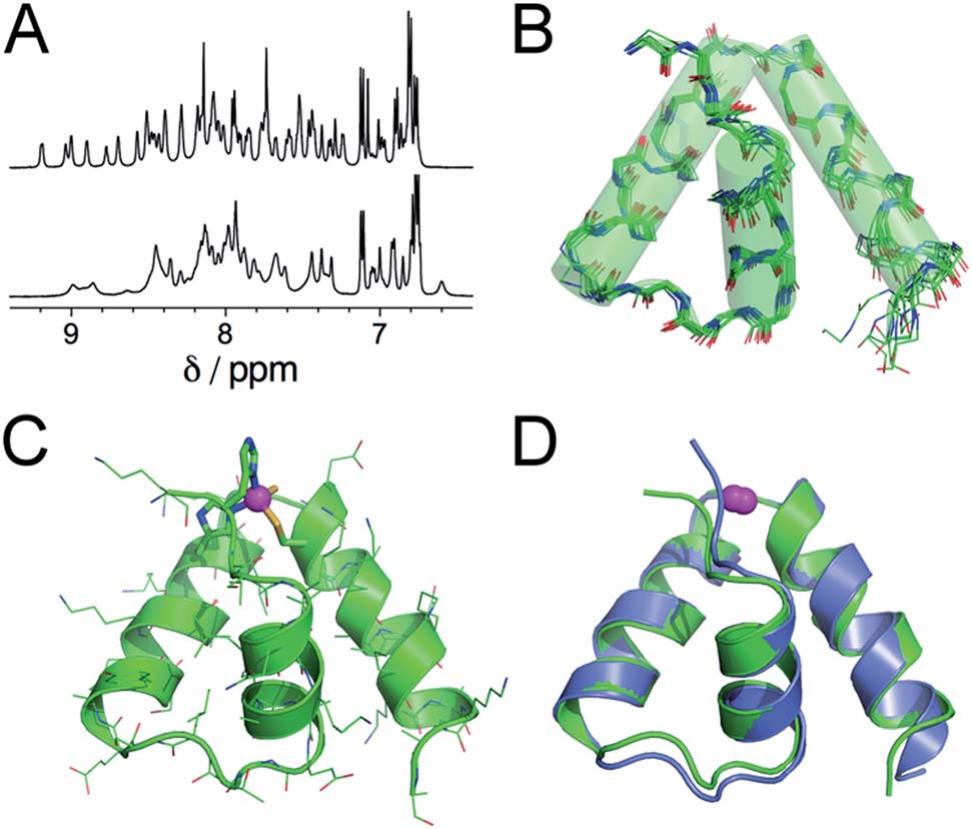
(A) ^1^H NMR spectra (500 MHz, H_2_O/D_2_O 9 : 1 pH 6.4, 298 K) of Zn·L_ASD_(HHCC) (top) and Zn·L_ASD_(AHCC) (bottom). (B) Superimposition of the 10 lowest energy structures of Zn·L_ASD_(HHCC) calculated using X-PLOR with NMR-derived distance and dihedral angle constraints. (C) Lowest energy structure of Zn·L_ASD_(HHCC). (D) Superimposition of the solution structure of Zn·L_ASD_(HHCC) (green) and the corresponding sub-domain of the crystallographic structure of ChrR (blue, pdb ; 2Z2S).^10^ The Zn^2+^ ion is shown in magenta.

Further insights into the structure of Zn·L_ASD_(HHCC) and Zn·L_ASD_(AHCC) were obtained by ^1^H NMR. The 1D ^1^H NMR spectra of metal-free L_ASD_(HHCC) and L_ASD_(AHCC) in H_2_O/D_2_O 9 : 1 display broad peaks with amide NH in the range 7.2–8.5 ppm indicating that these peptides are mostly random coil, in agreement with CD. Regarding Zn^2+^ complexes, the ^1^H NMR spectrum of Zn·L_ASD_(HHCC) displays sharp amide NH resonances spread over a wider range from 6.9 to 9.3 ppm (Fig. 4A, top, and ESI†). Many of them present ^3^*J*_HN,Hα_ < 6 Hz indicative of helical folding (Table S2 and Fig. S4 of ESI†). The 2D NOESY spectrum shows numerous correlation peaks corresponding to non-sequential NOEs that are characteristic of helical folding (Fig. S4 of ESI†). Additionally, several long-range NOEs between hydrophobic amino acid remote in the sequence indicate the formation of a hydrophobic core. This suggests the formation of a Zn^2+^ complex with a well-defined stable conformation. On the contrary, the ^1^H NMR spectrum of Zn·L_ASD_(AHCC) (Fig. 4A, bottom) shows very broad resonances in the amide NH region although this complex displays a similar helix content as Zn·L_ASD_(HHCC) as judged from its CD spectrum. This suggests a conformational equilibrium for Zn·L_ASD_(AHCC). Indeed, the coordination of the N-terminal histidine to the Zn^2+^ ion is not necessary to fold the three helices, as indicated by CD but it plays a major role in freezing the conformation of the peptide in HHCC variant. Finally, the structure of Zn·L_ASD_(HHCC) was calculated using X-PLOR with 466 H–H distance constraints (142 intraresidue, 138 sequential and 186 medium- and long-range) extracted from the NOESY spectrum and 35 *φ* dihedral constraints derived from ^3^*J*_HN,Hα_ values. The superimposition of the ten lowest energy structures, which is depicted on Fig. 4B shows that Zn·L_ASD_(HHCC) adopts a well-defined conformation with three helices (the backbone root mean square deviation over the ten structures is 0.83 Å). The zinc finger site caps this three-helix domain (Fig. 4C). The superimposition of the lowest energy structure of Zn·L_ASD_(HHCC) with the zinc finger site taken from the crystallographic structure of ChrR anti-sigma factor domain (Fig. 4D) shows that the model reproduces almost perfectly the fold of the native protein, including helices H1, H2 and H3 as well as the loops between the helices. To summarize, L_ASD_(HHCC) is able to bind only one Zn^2+^ ion to form a tetrahedral Zn(Cys)_2_(His)_2_ site that folds the peptide into a unique 3-helix conformation reproducing almost perfectly that of ChrR anti-sigma factor domain.

### Oxidation of Zn·L_ASD_(HHCC) by H_2_O_2_

The reactivity of Zn·L_ASD_(HHCC) toward H_2_O_2_ and ^1^O_2_ was investigated in order to assess the propensity of the anti-sigma factor domain zinc finger site to be oxidized by these two oxidants in comparison with other zinc fingers. The reaction of Zn·L_ASD_(HHCC) (20 μM) with H_2_O_2_ (1 mM) in phosphate buffer (10 mM, pH 7.0) was monitored by CD (Fig. S3C of ESI†). This reaction is slow and after 15 h, the CD spectrum resembles that of zinc-free L_ASD_(HHCC), indicating that the peptide unfolds upon reaction with H_2_O_2_. The product of the reaction was identified by ESI/MS as a disulfide (loss of two mass units). A similar CD spectrum was obtained by reacting Zn·L_ASD_(HHCC) with 2.5 eq. HOCl, a more efficient oxidant for zinc-bound thiolates known to form disulfides.^34^ The kinetics of the reaction of Zn·L_ASD_(HHCC) with H_2_O_2_ at 298 K was determined using previously described procedures,^19^ monitoring either the loss of the LMCT absorption in the UV or zinc release by using 4-(2-pyridylazo)resorcinol (PAR), which forms the Zn(PAR)_2_ complex with intense absorption in the visible when Zn^2+^ is released from the peptide upon oxidation. The PAR assay is not recommended to follow the oxidation of Zn(Cys)_3_(His) or Zn(Cys)_4_ because partially oxidized peptides displaying a reduced cysteine may retain Zn^2+^,^19^ but for this Zn(Cys)_2_(His)_2_ zinc finger peptide, both methods gave the same result. In condition of excess H_2_O_2_, a mono-exponential evolution of the absorption signal (either LMCT or Zn(PAR)_2_ band at 220 and 494 nm, respectively) is observed, indicating an apparent pseudo-first order reaction. Varying the concentration of H_2_O_2_ revealed a linear dependence on [H_2_O_2_] of the apparent first-order rate constant *k*^obs^ (Fig. S3 of ESI†). Thus, the rate-determining step of the reaction is second order, first order in H_2_O_2_ and first order in Zn·L_ASD_(HHCC), as previously observed for other zinc fingers.^18^–^20^ Indeed, the rate-determining step corresponds to the nucleophilic attack of H_2_O_2_ by the zinc-bound thiolate and its rate is given by *r* = *k* × [H_2_O_2_][Zn·L_ASD_(HHCC)] with *k* = 0.030 ± 0.002 M^–1^ s^–1^ at 297 K.

### Oxidation of Zn·L_ASD_(HHCC) by ^1^O_2_

**Fig. 5:**
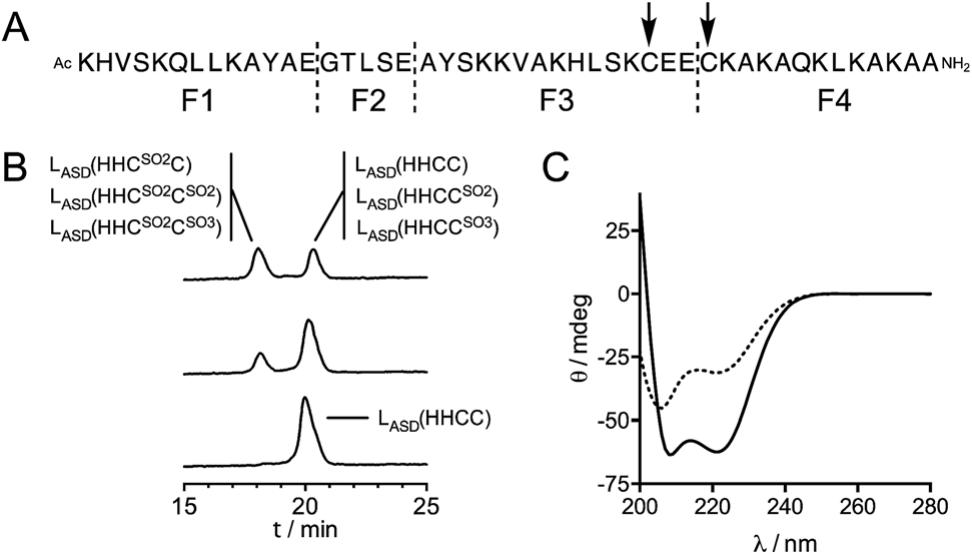
(A) GluC digestion sites (dashed lines) and ^1^O_2_ oxidation sites (arrows) identified for L_ASD_(HHCC). (B) HPLC chromatograms obtained before (bottom) and after 1 min (middle) and 5 min (top) irradiation of a solution of Zn·L_ASD_(HHCC) (60 μM) containing rose bengal (2 μM), in 20 mM Pi (D_2_O pD 7.0). The species identified by digestion and ESI-MS analysis of each collected peak are displayed. (C) CD spectra before (solid line) and after 10 min irradiation (dotted line) of a solution of Zn·L_ASD_(HHCC) (30 μM) containing rose bengal (3 μM), in 20 mM Pi (H_2_O pH 7.0).

In previous studies,^21^,^22^ we have shown that the reaction of Zn(Cys)_4_ and Zn(Cys)_2_(His)_2_ zinc finger models with ^1^O_2_ yields sulfinate species as major products, and disulfides in a lesser extent. Additionally, in a classical ββα Zn(Cys)_2_(His)_2_ zinc finger, Zn^2+^ coordination inhibits photooxidation of histidines. Oxidation of Zn·L_ASD_(HHCC) by ^1^O_2_ was investigated as previously described for other Zn(Cys)_4_ and Zn(Cys)_2_(His)_2_ zinc finger models:^21^,^22^ the oxidation products were identified by combination of HPLC and ESI-MS analyses and the reaction rate was assessed in competition experiments with a reference compound. Rose bengal or methylene blue were used as photosensitizers to produce ^1^O_2_ in this study. Zn·L_ASD_(HHCC) was photooxidized in D_2_O buffered with phosphate or ammonium acetate. HPLC analyses were performed with acidic eluent (0.1% TFA) so that Zn^2+^ is removed from the peptide during analysis. The HPLC chromatogram of a solution of Zn·L_ASD_(HHCC) containing the photosensitizer but maintained in the dark showed a single peak at *t*_R_ = 20.3 min corresponding to the reduced peptide L_ASD_(HHCC) (Fig. 5B). Upon irradiation, a second peak appears at a shorter retention time (*t*_R_ = 18.1 min). Prolongated irradiation shows an increase of the peak at 18.1 min at the expense of the one at 20.3 min. The main product detected by ESI-MS analysis of the crude corresponds to the addition of two oxygen atoms to the peptide, suggesting the formation of sulfinates. Other peaks corresponding to the addition of three and four oxygen atoms are also observed. The two HPLC peaks were collected, digested with glutamate carboxypeptidase (GluC, a peptidase that cleaves peptides at carboxylic side of glutamates or aspartates), and digestion mixtures were analysed by ESI-MS (Fig. 5A and Table S1†). GluC digestion of L_ASD_(HHCC) can give four fragments: Ac-KHVSKQLLKAYAE (F1), GTLSE (F2), AYSKKVAKHLSKC^31^E(E) (F3) and C^34^KAKAQKLKAKAA-NH_2_ (F4). Digestion of both HPLC fractions gave unaltered N-terminal fragments F1 and F2, meaning that neither His2 nor Tyr11 are photooxidized. However, different patterns were observed for the two cysteine-containing fragments F3 and F4 (Table S1 of ESI†). For both HPLC fractions, no mass peak corresponding to the possible F3–F4 disulfide was detected. For the fraction eluting at 20.3 min, fragment F3 is unaltered but fragment F4 was detected in three distinct chemical forms: unaltered and with increase of 32 and 48 mass units. The product with a 32 mass unit increase was ascribed to the oxidation of Cys34 into a sulfinate by comparison to previous studies.^21^,^22^ This was confirmed by the loss of 66 mass units corresponding to H_2_SO_2_ upon MS/MS fragmentation. The formation of a sulfinate upon photooxidation of metal-bound thiolate has already been reported in many instances for Pt,^35^–^37^ Ni,^38^ Pd,^38^ Co^39^–^41^ and Cd^42^ complexes thereby supporting the formation of a sulfinate for Zn-bond cysteines in Zn·L_ASD_(HHCC) but the product with a 48-mass unit increase is more intriguing. As F4 does not contain any amino acid prone to oxygen incorporation upon photooxidation other than Cys34 (*i.e.* His, Tyr, Trp or Met), this product could only be ascribed to the formation of a sulfonate on Cys34. This hypothesis is supported by the formation of glutathione sulfonate in the case of photooxidation of glutathione.^43^ Nevertheless, the mechanism of the formation of this product, especially the breaking of the O–O bond in the putative RS(O)(O)–OO^–^ intermediate remains unclear. Altogether, the fraction eluting at 20.3 min contains the unoxidized peptide L_ASD_(HHCC) as expected but also L_ASD_(HHCC^SO_2_^) and L_ASD_(HHCC^SO_3_^) oxidation products in which Cys34 was photooxidized into a sulfinate and a sulfonate, respectively (Fig. 5A). For the fraction eluting at 18.1 min, only a 32-mass unit increase was detected for F3, revealing photooxidation of Cys31 into a sulfinate (confirmed by loss of 66 mass units upon MS/MS fragmentation). Photooxidation of Cys31 rather than Tyr20 or His27 in F3 was supported by unaltered F1 fragment, which contains also histidine and tyrosine, and was further demonstrated by the loss of 66 Da corresponding to H_2_SO_2_ upon MS–MS fragmentation. F4 appears within three different forms: unaltered and with +32 and +48 mass units. This indicates the presence of Cys34 in reduced, sulfinate and sulfonate forms in the fraction eluting at 18.1 min. Therefore, this fraction contains three peptides: the primary oxidation product L_ASD_(HHC^SO_2_^C) and the overoxidation products L_ASD_(HHC^SO_2_^C^SO_2_^) and L_ASD_(HHC^SO_2_^C^SO_3_^) (Fig. 5B). Noteworthy, mass analysis always revealed the presence of overoxidation products, even at short irradiation times, indicating that the rate of formation of overoxidation products is at least comparable to the rate of formation of primary oxidation products L_ASD_(HHCC^SO_2_^) and L_ASD_(HHC^SO_2_^C).

Then, the rate of chemical reaction between Zn·L_ASD_(HHCC) and ^1^O_2_ (*k*_r_) was assessed by competitive photooxidation with the peptide EGWGK as a competitor (*k*_r_(EGWGK) = (4.6 ± 0.5) × 10^6^ M^–1^ s^–1^)^21^ following a procedure described previously.^21^,^22^ The method consists in comparing the consumption of the zinc finger peptide and the reference compound by HPLC (ESI). In such a competitive photooxidation experiment, the ratio of the rate constants *k*_r1_ and *k*_r2_ of two compounds C_1_ and C_2_ is given by eqn (1) where [C_*i*_]_0_ and [C_*i*_]_f_ are the concentrations of compound C_*i*_ before and after photooxidation, respectively.^44^,^45^
1
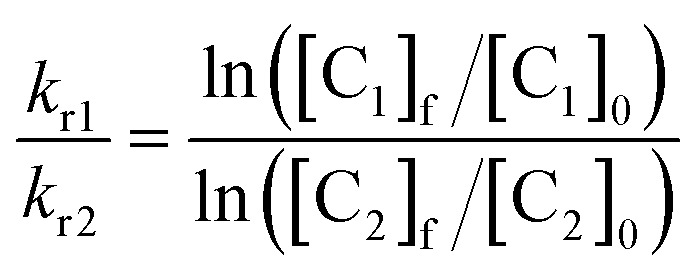



Since we were not able to separate unreacted L_ASD_(HHCC) and L_ASD_(HHCC^SO_2_^) by HPLC and as overoxidation are rapidly formed, this method underestimates the consumption of L_ASD_(HHCC) and yields a sizeable underestimation of *k*_r_. Nevertheless, a lower limit of 3.9 × 10^6^ M^–1^ s^–1^ for *k*_r_(Zn·L_ASD_(HHCC)) was calculated from the competition experiments. Finally, CD was used to assess the consequences of ^1^O_2_ oxidation of Zn·L_ASD_(HHCC) on the peptide fold. As shown in Fig. 5C, photooxidation causes dramatic loss of helical structure, attesting of peptide unfolding.

## Discussion

### Design and characterization of the anti-sigma factor domain zinc finger model

For several years, we have been developing peptidic zinc finger models of small size in order to study the reactivity of zinc finger sites toward reactive oxygen species such as H_2_O_2_,^18^–^20^ HOCl^34^ or ^1^O_2_ 21,22 at the molecular level. Our approach involves the use of cyclic and branched peptides, named CPLT (Cyclic Peptides with Linear Tail),^29^ to create shortcuts in native zinc finger sequences. This affords models that reproduce almost perfectly the fold of native zinc finger domains around the Zn^2+^ ion with a minimal set of amino acids. The small size of these models allowed us to characterize oxidation products and to provide reliable kinetic data to describe the reactivity of zinc fingers. The CPLT design is well suited to zinc finger sites harbouring a CXXC motif in a β-hairpin loop. However, the elaboration of a CPLT model was not possible for the anti-sigma factor domain due to its 3-helix core that supports the four Zn^2+^-binding amino acids. Therefore, we decided to keep this core but to reduce the size of the anti-sigma factor domain sequence as much as possible without altering its fold around Zn^2+^. Re-engineering of the amino acid sequence allowed us to get a soluble 46-amino acid protein that folds upon Zn^2+^ binding and adopts the same conformation as that found in the sigma factor/anti-sigma factor complex. The design strategy used to elaborate this model revealed some important features: (i) it is necessary to avoid clustering of negatively or positively charged amino acids on the surface to provide sufficient solubility to the model, (ii) once the Zn^2+^ ion coordinated to the H–X_3_–C–X_2_–C motif, the constitution of a hydrophobic core within the heart of the 3-helix structure is most probably the driving force for the protein to get the proper folding; and (iii) the N-terminal histidine is absolutely required for conformational stability, providing at least 4 kcal mol^–1^ stabilization to the system as shown by the comparison between Zn·L_ASD_(HHCC) and Zn·L_ASD_(AHCC).

### H_2_O_2_ and ^1^O_2_ oxidation

In previous studies on the oxidation of model zinc fingers, we have shown that H_2_O_2_ yields mainly disulfides as products at physiological pH conditions whereas ^1^O_2_ yields mainly sulfinates. Regarding kinetics, we have observed that neutral Zn(Cys)_2_(His)_2_ zinc finger sites react slower with H_2_O_2_ than negatively charged Zn(Cys)_3_(His) and Zn(Cys)_4_ zinc finger sites.^19^,^20^ The reactivity of Zn·L_ASD_(HHCC) toward H_2_O_2_ was studied using the methodology previously described.^19^ Reaction with H_2_O_2_ leads to disulfide formation within the CEEC motif with a second order rate constant of 0.030 ± 0.002 M^–1^ s^–1^ at 297 K. This is within the range of second order rate constants determined for other Zn(Cys)_2_(His)_2_ zinc fingers (*k* = 0.008–0.037 M^–1^ s^–1^) but significantly lower than for Zn(Cys)_3_(His) and Zn(Cys)_4_ zinc fingers (*k* = 0.1–1.5 M^–1^ s^–1^), confirming the trend previously observed.^20^ Among Zn(Cys)_2_(His)_2_ zinc fingers models, Zn·L_ASD_(HHCC) reacts faster with H_2_O_2_ than Zn·CP-1(CCHH) (*k* = 0.008 M^–1^ s^–1^), a classical CCHH zinc finger with well-defined ββα fold. Regarding ^1^O_2_, reaction of Zn·L_ASD_(HHCC) is very fast leading to sulfinates but overoxidation products (sulfonate, bis-sulfinate) are also formed very rapidly. No photooxidation of Zn^2+^-bound histidines was detected, confirming that histidines are protected from ^1^O_2_ oxidation by Zn^2+^ coordination, as previously observed in the case of a classical ββα zinc finger.^22^ Unfortunately, all photooxidation products could not be separated by HPLC precluding precise determination of the chemical reaction rate constant *k*_r_.^21^,^22^ This was not the case in previous studies. Indeed, the size of the Zn·L_ASD_(HHCC) model is large compared to our other previously described models (46 *versus ca.* 25 amino acids), rendering HPLC separation less sensitive to changes occurring at a single amino acid position. Hence, the size of the model clearly matters for optimal separation and the smaller the model, the more detailed the molecular information. Nevertheless, a lower limit of 3.9 × 10^6^ M^–1^ s^–1^ could be determined for *k*_r_. The observation of at least two overoxidation products – even at short irradiation time – in the peak eluting at 20.3 min (Fig. 5A) during analysis of photooxidation products of Zn·L_ASD_(HHCC) indicates that this *k*_r_ value is probably underestimated. Hence, Zn·L_ASD_(HHCC) reacts with ^1^O_2_ at least as fast as, but most probably faster than, Zn·L_TC_ (*k*_r_ = (4.3 ± 0.4) × 10^6^ M^–1^ s^–1^), a Zn(Cys)_4_ treble clef zinc finger model, on contrary to what is observed for H_2_O_2_. In the case of H_2_O_2_, negatively charged Zn(Cys)_4_ sites react faster than neutral Zn(Cys)_2_(His)_2_ sites. Therefore, the observed trend for H_2_O_2_ regarding the charge of the zinc finger site and its reactivity cannot be extrapolated to ^1^O_2_ reactivity. The lower limit value of *k*_r_ determined for Zn·L_ASD_(HHCC) is higher than the *k*_r_ value determined for Zn·CPF (*k*_r_ = (0.70 ± 0.07) × 10^6^ M^–1^ s^–1^), a model peptide for the CCHH classical ββα zinc fingers, which differs from Zn·CP-1(CCHH) by a single Tyr to Phe mutation only. In order to understand the greater reactivity of Zn·L_ASD_(HHCC) compared to the classical ββα zinc finger CP-1/CPF observed for H_2_O_2_ and ^1^O_2_, we have examined the structure of the Zn(Cys)_2_(His)_2_ sites in both models focusing on two important factors for cysteine sulfur reactivity: solvent accessible surface and number of NH···S hydrogen bonds, the latter being known to decrease the nucleophilic reactivity of Zn^2+^-bound sulfurs.^23^,^46^ Based on our NMR structure of Zn·L_ASD_(HHCC) and the pdb file ; 1MEY 47 for Zn·CP-1(CCHH), we found that the former has higher sulfur solvent accessible surface area (8.8 Å^2^*vs.* 4.1 Å^2^) and less NH···S hydrogen bonds (1 *vs.* 3). Therefore, these two factors support the greater reactivity of the anti-sigma factor domain zinc finger model compared to the classical ββα model.

### Biological relevance

Zinc binding to ChrR and formation of the Zn(Cys)_2_(His)_2_ zinc finger site of ChrR is needed to ensure its proper folding and sequestering of σ^E^.^8^ Plus, it was demonstrated that the anti-sigma factor domain of ChrR is sufficient to sequester σ^E^.^10^ Our data show that the Zn·L_ASD_(HHCC) model can be oxidized by ^1^O_2_ leading to its unfolding. This supports the hypothesis that photooxidation of the cysteines of ChrR Zn(Cys)_2_(His)_2_ zinc finger site may be a key event in the ^1^O_2_ sensing mechanism by ChrR promoting the breakdown the ChrR–σ^E^ complex. Nevertheless, to be a sensing unit this zinc finger site would require fast reaction with ^1^O_2_ in order to trigger quickly the cellular response against ^1^O_2_ before too many oxidative damages are produced. The kinetic data gained in this study are also in favour of a sensing role for ChrR Zn(Cys)_2_(His)_2_ site: it reacts with ^1^O_2_ faster than other known structural zinc finger sites, either of the Zn(Cys)_2_(His)_2_ 22 or Zn(Cys)_4_ 21 type. Indeed, it seems that the uncommon topology of the zinc finger site of ChrR^12^ provides very high zinc affinity while maintaining “naked” cysteinates, with a low number of NH···S hydrogen bonds, for higher reactivity. However, Rajasekar *et al.* have shown that oxidation of the HHCC zinc finger of ZAS proteins is not always sufficient to dissociate the anti-sigma factor from its cognate sigma factor.^17^ In the case of ChrR, it was clearly demonstrated that its C-terminal cupin-like domain is required for the ^1^O_2_ response.^10^,^11^,^17^ Therefore, it is possible that ^1^O_2_ oxidation of both the zinc finger of ChrR anti-sigma factor domain and essential histidine residues of the cupin-like domain are required to cause sufficient conformational change to allow the dissociation of the ChrR–σ^E^ complex. This would correspond to a dual activation process making this system responsive to ^1^O_2_ only among ROS, for a specific triggering of the transcriptional response. Alternatively, the essential histidine and glutamate residues of the cupin-like domain (H141, H143, E147 and H177) could provide alternative Zn^2+^ ligands to compensate for the oxidized cysteines of the anti-sigma factor domain. A mixed coordination set involving residues of both the anti-sigma factor domain and the cupin-like domain around Zn^2+^ would generate sufficient conformational rearrangement to destabilize the ChrR–σ^E^ complex.

## Conclusions

In this article, we have described the elaboration of a model for the Zn(Cys)_2_(His)_2_ zinc finger site of the anti-sigma factor domain of ChrR, a protein involved in transcriptional response to ^1^O_2_ in several bacteria. This site has a special topology among zinc fingers and its role in ^1^O_2_ sensing by ChrR is under debate. This model, namely Zn·L_ASD_(HHCC), is a 46-amino acid peptide inspired from ChrR anti-sigma factor domain sequence. It forms a 3-helix core when Zn^2+^ binds to the four cysteine and histidine side chains. Zn·L_ASD_(HHCC) adopts the same structure as the corresponding sequence in ChrR anti-sigma factor domain. We have shown that the cysteines of the Zn(Cys)_2_(His)_2_ site of Zn·L_ASD_(HHCC) are oxidized by ^1^O_2_ into sulfinates and other overoxidation products. Photooxidation is faster than that of other zinc finger sites, especially compared to classical ββα Zn(Cys)_2_(His)_2_ zinc fingers. Additionally, ^1^O_2_ oxidation destabilizes the zinc finger leading to its unfolding. The data presented in this work indicate that photooxidation of the zinc finger site of ChrR may be a key event in its ^1^O_2_ sensing mechanism.

## Conflicts of interest

There are no conflicts to declare.

## Supplementary Material

Supplementary information
