# Supplementary material for: Model peptide for anti-sigma factor domain HHCC zinc fingers: high reactivity toward 1O2 leads to domain unfolding
Source: Chem Sci. 2019 Feb 21;10(12):3608–15. doi: 10.1039/c9sc00341j (PMC6432622; doi:10.1039/c9sc00341j)
Supplement: Supplementary file 1 [file SC-010-C9SC00341J-s001.pdf]

Electronic Supplementary Material (ESI) for Chemical Science.
This journal is © The Royal Society of Chemistry 2019

Model Peptide for Anti-Sigma Factor Domain HHCC Zinc Fingers: High Reactivity toward $^1O_2$ Leads to Domain Unfolding

Valentin Chabert,[a] Vincent Lebrun,[a] Colette Lebrun,[b] Jean-Marc Latour,[a] and Olivier Sénèque*[a]

[a] Univ. Grenoble Alpes, CNRS, CEA, BIG, LCBM (UMR 5249), F-38000 Grenoble, France.

[b] Univ. Grenoble Alpes, CEA, INAC, SyMMES, F-38000 Grenoble, France.

Email: *olivier.seneque@cea.fr*

Supporting Information

Content

Abbreviations 2
Materials and methods 2
Peptide synthesis 2
Preparation of zinc finger stock solutions 3
Absorption and circular dichroism 3
Oxidation by H_2O_2 4
Oxidation by $^1O_2$ 5
Solution structure determination 6
References 13

Abbreviations

PyBOP: (Benzotriazol-1-yloxy)tripyrrolidino-phosphonium-hexafluorophosphate; $Pd(PPh_3)_4$: *tetrakis*(triphenyl-phosphine)-palladium(0); TIS: triisopropylsilane; TCEP: *tris*(2-carboxyethyl)phosphine; DIEA: N,N-diisopropylethylamine; TFA: trifluoroacetic acid; MeOH: methanol; DCM: dicholoromethane; Et_2O: diethylether; DMF: N,N-dimethylformamide; *t*Bu: *tert*-butyl; Trt: trityl; Fmoc: 9-fluorenylmethoxycarbonyl; Boc: *tert*-butyloxycarbobyl; EDTA: ethylenediamine-tetraacetic acid; HPLC: high performance liquid chromatography; ESI: electrospray ionization; MS: mass spectrometry; UV-Vis: ultraviolet-visible; CD: circular dichroism.

Materials and methods

Reagents and solvents: N-α-Fmoc-protected amino acids for peptide synthesis, PyBOP coupling reagent and NovaPEG Rink Amide resin were obtained from Novabiochem. Other reagents for peptide synthesis, solvents, buffers and metal salts were purchased from Sigma-Aldrich. All buffers or metal solutions were prepared with MilliQ water (Millipore). Buffer solutions were treated with Chelex 100 resin (Biorad) to remove trace metal ions. The concentration of the Zn^{2+} and Co^{2+} stock solutions was determined by colorimetric EDTA titrations.[1]

Analyses and purifications: RP-HPLC was used for analyses and purifications of the peptides. Mixtures of solvent A (0.1% TFA in H_2O) and B (0.1% TFA in MeOH/H_2O 9:1) were used as mobile phase. Analytical HPLC separations were performed on an Agilent Infinity 1200 system using Merck Chromolith RP-18e (150 mm × 4.6 mm) columns. Method A consisted in 5% B during 2 min, a 5 to 55 % B linear gradient in 5 min, then a 55 to 90 % B linear gradient in 15 min at 2 mL/min. Preparative HPLC separations were performed on a VWR LaPrepΣ system using a Waters XBridge Peptide BEH130 C18 (5 µm, 150 mm × 19 mm) column at 10 mL/min. The preparative separation method consisted in 5% B during 2 min, a 5 to 50 % B linear gradient in 5 min then a 50 to 90 % B linear gradient in 25 min at 10 mL/min. Eluate was monitored by electronic absorption at 214, 254 and 280. ESI-MS analyses were performed on a Thermo LXQ spectrometer. UV-Vis absorption spectra were recorded on a Perkin-Elmer Lambda 35 spectrophotometer or on a Varian Cary 50 spectrophotometer. Circular dichroism spectra were recorded on an Applied Photophysics Chirascan spectropolarimeter.

Peptide synthesis

Elongation of L$_{\mathrm{ASD}}$(**X**HCC) Ac-K**X**VSKQLLKAYAEGTLSEAYSKKVAKHLSKCEECKAKAQKLKAKAA-NH_2 (with **X** = H or A) was performed on NovaPEG Rink Amide resin (0.40 mol g^{-1}; 0.13 mmol scale) using standard SPPS protocols using Fmoc/tBu chemistry on an automated peptide synthesizer (CEM Liberty1 Microwave Peptide Synthesizer) after attachment of the first amino acid by single manual coupling (30 min) using 2-fold excess of Fmoc-Gly-OH, 2-fold excess of PyBOP and 6-fold excess of DIEA in DMF followed by acetylation using Ac_2O/pyridine/DMF (1:2:7 (by vol.), 10 mL, 5 min). For automated synthesis, single coupling (5 min, 50°C, 25 W microwave power) were performed using 4-fold molar excess of Fmoc-L-amino acid, 4-fold molar excess of PyBOP and 8-fold molar excess of DIEA. A capping step was performed after each coupling with Ac_2O/DIEA in DMF (5 min, 65 °C, 40 W microwave power). Fmoc removal was performed using 20% piperidine in DMF (30 s + 3 min, 70°C, 40 W microwave power). Dipeptides underlined in the above sequence were introduced as pseudo-proline dipeptides (Fmoc-Val-Ser($\Psi^{Me,Me}$pro)-OH, Fmoc-Gly-Thr($\Psi^{Me,Me}$pro)-OH, Fmoc-Tyr(*t*Bu)-Ser($\Psi^{Me,Me}$pro)-OH and Fmoc-Leu-Ser($\Psi^{Me,Me}$pro)-OH) by manual coupling as describe above for the

first amino acid. Before the last two amino acids, the resin was divided into two parts in a 6:1 ratio. The KA terminus was assembled on the small batch and the KH terminus on the biggest. The N-terminus was acetylated manually using Ac_2O/pyridine/DMF (1:2:7 (by vol.), 10 mL, 5 min). Removal of acid-labile protecting groups and resin cleavage were performed using TFA/H_2O/TIS/DTT (19 mL:0.6 mL:0.6 mL:600 mg) for 2 h. TFA was evaporated under reduced pressure and cold Et_2O was added to precipitate the peptide. The pure peptide was obtained after HPLC purification and freeze-drying. **LASD(HHCC):** Yield = 13 % (98 mg); HPLC (anal.) t_R = 14.7 min (method A); ESI-MS: average *m/z* = 1272.7 (4+), 1018.3 (5+), 848.8 (6+), 727.8 (7+), 636.9 (8+), 566.3 (9+), 509.8 (10+) / calculated av. *m/z* = 1272.75 $[M+4H]^{4+}$, 1018.40 $[M+5H]^{5+}$, 848.84 $[M+6H]^{6+}$, 727.72 $[M+7H]^{7+}$, 636.88 $[M+8H]^{8+}$, 566.23 $[M+9H]^{9+}$, 509.71 $[M+10H]^{10+}$ for M = $C_{225}H_{383}N_{65}O_{64}S_2$). **LASD(AHCC):** Yield = 13 % (16 mg); HPLC (anal.) t_R = 16.6 min (method A); ESI-MS: average *m/z* = 1256.1 (4+), 1005.0 (5+), 837.7 (6+), 718.3 (7+), 628.7 (8+), 558.9 (9+) / calculated av. *m/z* = 1256.24 $[M+4H]^{4+}$, 1005.19 $[M+5H]^{5+}$, 837.83 $[M+6H]^{6+}$, 718.28 $[M+7H]^{7+}$, 628.62 $[M+8H]^{8+}$, 558.89 $[M+9H]^{9+}$ for M = $C_{222}H_{381}N_{63}O_{64}S_2$).

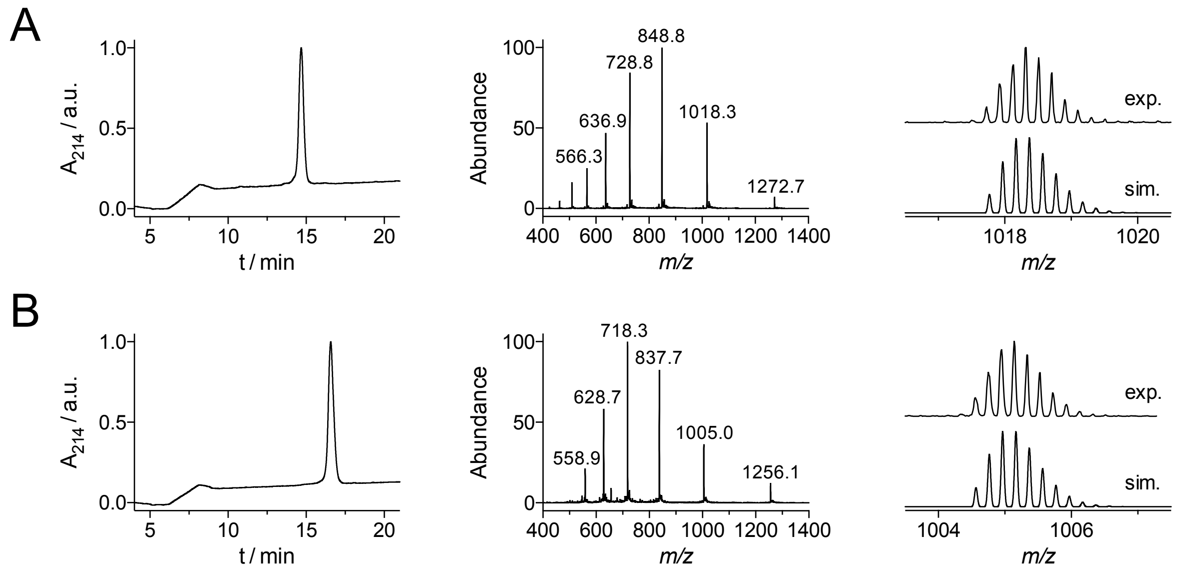

Fig. S1 HPLC chromatograms (*left*) and ESI-MS spectra (*middle*: full MS spectrum; *right*: experimental and simulated isotopic pattern of the $[M+5H]^{5+}$ peak) of (A) LASD(HHCC) and (B) LASD(AHCC).

Preparation of zinc finger stock solutions

Solutions of metal-free LASD peptides (*ca.* 1 mM) were prepared by dissolution of the lyophilized peptides in H_2O (or D_2O for photooxidation experiments) under argon atmosphere. The exact peptide concentration was determined by Ellman's assay.[2] Solutions of Zn·LASD (*ca.* 1 mM) complexes were prepared by adding 1.1 eq. of Zn^{2+} dissolved H_2O (100 mM). The pH was adjusted to 7.0 using NaOH (or NaOD).

Absorption and circular dichroism

A solution of metal-free LASD peptide (10-20 µM and 100-150 µM for Zn^{2+} and Co^{2+} titrations, respectively) in phosphate buffer (20 mM, pH 7.0) containing TCEP (250 µM) was prepared from stock solutions of peptide, phosphate buffer (100 mM, pH 7.0) and TCEP (33 mM). Titrations were performed at 298 K under argon by adding aliquots of a degassed metal stock solution to a rubber-sealed quartz cell (0.4 cm or 1 cm path length) containing the peptide solution. UV-Vis spectra were recorded every 1 nm at a scan rate of 240 nm/min. CD

spectra were recorded from 300 nm to 200 nm every 1 nm with a 2 s signal averaging for each point. Each spectrum was recorded twice, averaged and smoothed using a Savitzky-Golay filter.

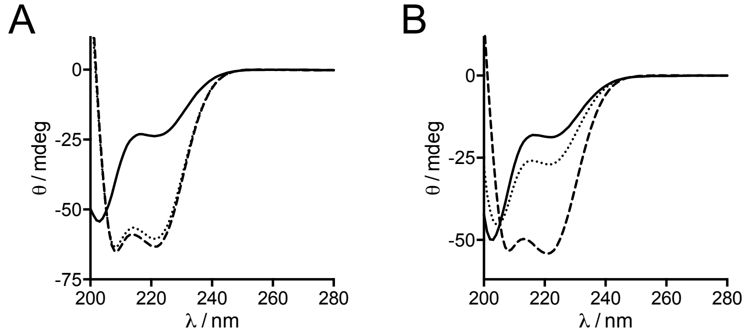

Fig. S2 Assessment of Zn^{2+} binding constants by competition experiments. CD spectra of solutions containing LASD(HHCC) (A) or LASD(AHCC) (B) and one equivalent of EDTA in phosphate buffer (10 mM, pH 7.0) before addition of Zn^{2+} (solid lines) and after addition of 1.0 eq. Zn^{2+} (dotted lines) and 2.0 eq. Zn^{2+} (dashed lines).

Oxidation by H_2O_2

A solution of Zn·LASD(HHCC) (20 μM) and H_2O_2 (5-35 mM) was prepared in an appropriate buffer (pH 7.0) and the reaction was monitored by absorption spectrophotometry. Based on previous work, the decay of the LMCT band at 220 nm was used to monitor the reaction in phosphate buffer (100 mM).[3] The kinetic traces (Fig. S3A) could be fitted by mono-exponential to yield the apparent first-order rate constants, k^{obs}. Fig. S3B shows the linear dependence of k^{obs} against [H_2O_2], which yielded k = 0.031 ± 0.003 M^{-1} s^{-1}. Fig. S3C shows the CD spectra recorded before introduction of H_2O_2 (1 mM) and after reaction (15 h). The PAR assay was thus used to confirm the value of k.[3] Oxidations were conducted in presence of PAR (100 μM) in Bis-Tris buffer 1(00 mM) and the appearance of $Zn(PAR)_2$ absorption at 494 nm was recorded. Mono-exponential fit of the kinetic traces yielded k^{obs} values, which were plotted against [H_2O_2] to yield k = 0.030 ± 0.001 M^{-1} s^{-1} (Fig. S3D).

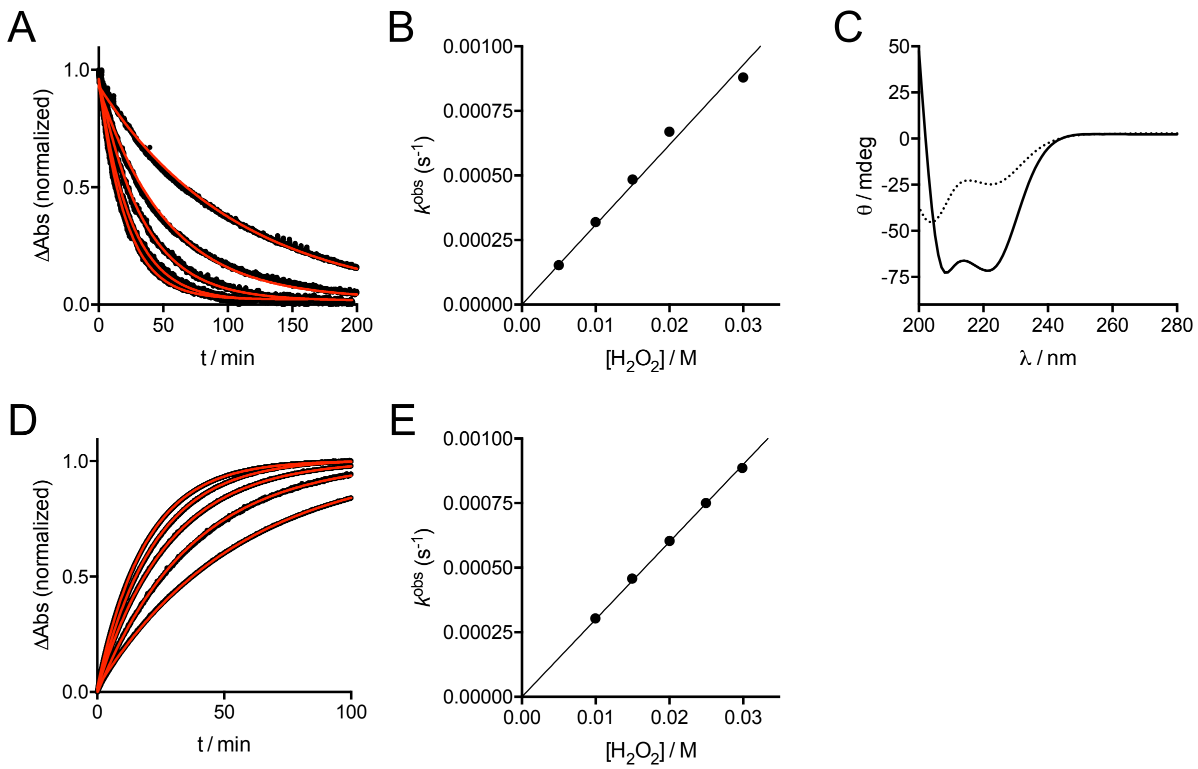

Fig. S3 Oxidation of Zn·L$_{ASD}$(HHCC) by H_2O_2. (A) Kinetic traces (black, normalized) for the LMCT monitoring (220 nm) with mono-exponential fits in red. (B) Plot of k^{obs} against [H_2O_2] for the LMCT monitoring. The solid line corresponds to the linear regression yielding $k = 0.031 \pm 0.003$ M^{-1} s^{-1}. (C) CD spectra recorded before (solid line) and after (dashed line) reaction with H_2O_2. (D) Kinetic traces (black, normalized) for the PAR monitoring (494 nm) with mono-exponential fits in red. (E) Plot of k^{obs} against [H_2O_2] for the PAR monitoring. The solid line corresponds to the linear regression yielding $k = 0.029 \pm 0.001$ M^{-1} s^{-1}.

Oxidation by $^1O_2$

Buffers: Deuterated buffer solutions were prepared by dissolving Na_2HPO_4 or ammonium acetate in D_2O (99,9% from Eurisotop, traces of metals removed by passing D_2O over CHELEX resin), and the pD was adjusted to 7.0 or 8.0 (with a regular pH meter, pD = pH$_{red}$ + 0.44)[4] with NaOD and DCl. Otherwise, buffer solutions were prepared in milliQ H_2O.

Photooxidation: Typical photooxidation experiments were performed as follows. In an Eppendorf tube, the solution of Zn·L$_{ASD}$(HHCC) in D_2O is diluted to 200 µM in buffer Pi 20 mM pD containing 1-3 µM photosensitizer (Rose Bengal or Methylene Blue). The sample is maintained at 278 K in a water-cooled bath in front of a 400 W halogen lamp. After *ca.* 5 min of temperature equilibration, irradiation is performed by turning the lamp on for 30 seconds to 10 minutes, depending on the sample and solvent (shorter irradiation times are required for D_2O solution compared to H_2O). Then, the sample (100-300 µL) is injected into the HPLC for analysis. CD monitoring of the photooxidation was performed using H_2O solutions.

Determination of the lower limit for the photo-oxidation rate k_r of Zn·L$_{ASD}$(HHCC): The competition experiments were conducted as previously described[5,6] using peptide EGWGK as a competitor. Photooxidation was performed following the photooxidation protocol described above, but in presence of methylene blue as photosensitizer and in 20 mM Pi pD 8.0. Consumption of the two competitors was monitored by integration of the

HPLC chromatograms obtained before and after irradiation. As noted in the text, the peak eluting at 20.3 min contains unreacted L_{ASD}(HHCC) as well as some oxidized peptides, but we considered that it contains L_{ASD}(HHCC) only. This led to underestimate the consumption of L_{ASD}(HHCC). The chemical reaction rate constant was calculated using the equation given in the article. Therefore, only a lower limit of k_r was obtained: k_r(Zn·L_{ASD}(HHCC)) < (3.9 ± 0.5)×10^6 M^{-1} s^{-1}.

ESI-MS analysis of photooxidation products: For ESI-MS analysis of the reaction mixture, Zn·L_{ASD}(HHCC) was photooxidized in ammonium acetate buffer (20 mM in D_2O, pD 7.0) and the solution was lyophilized. The resulting solid was dissolved in H_2O containing 0.1 % TFA and lyophilized three times for complete D/H exchange before ESI-MS analysis. Finally, the solid was dissolved in ammonium acetate (20 mM, pH 7.0) and analyzed by ESI-MS. For digestion experiments, products were separated by HPLC, collected and lyophilized. The two collected fractions (0.1-0.2 mg) were dissolved in ammonium acetate (20 mM, pH 7.0, 190 µL). Glutamate carboxypeptidase GluC (200 ng×µL^{-1}, 10 µL) was added and solutions were incubated overnight at 37°C before freeze-drying. Fractions were dissolved in 0.1% TFA in H_2O or ammonium acetate (with or without TCEP) and analyzed by ESI-MS.

Table S1 ESI-MS analysis of GluC digested HPLC fractions of the photooxidation products of Zn·L_{ASD}(HHCC).

Fragment	Mass / Da	Observed *m/z* / Da for $[M+nH]^{n+}$ or $[M–nH]^{n–}$ species	
		peak eluting at 18.1 min	peak eluting at 20.3 min
Ac-KHVSKQLLKAYAE			
unaltered	1555.87	1556.8 (1+), 779.3 (2+), 519.8 (3+),	1556.8 (1+), 779.6 (2+)
		1554.6 (1–), 776.8 (2–), 1668.8 (1–) ($M+CF_3COO^-$)	1554.6 (1–)
AYSKKVAKHLSKC_{31}EE			
unaltered (CysSH)	1719.90	–	861.1 (2+), 1718.8 (1–)
sulfinic acid ($CysSO_2H$)	1751.89	877.2 (2+), 585.3 (3+)	–
		1750.8 (1–)*, 874.8 (2–), 931.6 (2–) ($M+CF_3COO^-$)	
C_{34}KAKAQKLKAKAA-NH_2			
unaltered (CysSH)	1356.85	1357.6 (1+), 679.3 (2+)	1357.8 (1+), 679.6 (2+)
		1469.2 (1–) ($M+CF_3COO^-$)	1355.7 (1–)
sulfinic acid ($CysSO_2H$)	1388.83	1387.6 (1–)*	1387.5 (1–)
sulfonic acid ($CysSO_3H$)	1404.82	1403.5 (1–)	1403.6 (1–)

* loss of 66 mass units (H_2SO_2) in MS/MS fragmentation.

Solution structure determination

NMR spectroscopy: All NMR experiments were recorded on a 500 MHz Bruker AVANCE II spectrometer equipped with a BBI probe with a z-axis gradient field. Samples were prepared by adding 1.1 molar equivalent of $Zn(ClO_4)_2$ to the peptide (*ca.* 1.8 mM) in H_2O/D_2O 9:1. The pH was adjusted to 6.3 with NaOH and HCl. 1D ^1H NMR spectra were recorded with 12-ppm window and 32k data points in the time domain. 2D ^1H-^1H spectra were acquired with Watergate[7,8] solvent suppression in phase-sensitive mode using 4096×1024 matrices over a 5000 Hz spectral width. TOCSY experiments were recorded at 298 K and 283 K using a MLEV-17 spin-lock sequence with a 70 ms mixing time. NOESY experiments were recorded at 298 K with a 200 ms mixing time. DQF-COSY spectra were recorded at 298 K. All spectra were processed with TOPSPIN 3.2.

Structure calculations: Cross peaks in NOESY spectra were integrated using the program SPARKY 3.114[9] and converted to H-H distances. Tyr Hδ/Hε (2.44 Å), Leu Hγ/Hδ, Thr Hβ/Hγ, Val Hβ/Hγ (2.80 Å) and His Hε1/Hδ2 (4.21 Å) were used as references for distance calibrations. Multiplicity was taken into account. Upper distance restraints were set to 2.7 Å, 3.5 Å, 5.0 Å and 7.0 Å for distances ≤ 2.5 Å, ≤ 3.3 Å, ≤ 4.8 Å and ≥ 4.8 Å, respectively. $^3J_{HN,H\alpha}$ coupling constants were measured on 1D ^{1}H NMR spectra experiments. ϕ dihedral restraints were derived from $^3J_{HN,H\alpha}$. Non-stereospecifically assigned protons were treated as pseudo-atoms. All peptide bond ω angles were set to *trans*. Solution structures were calculated using the program X-PLOR 3.851[10] following standard refinement protocols starting from random structures with r^{-6} averaging. The non-bonded interactions were modeled by the PARALLHDG force field. A first calculation was performed not including the zinc ion in the topology file to determine the overall fold of the peptide. The structure revealed the folding of helix H1, H2 and H3 and their close packing. The side chains of the two His and the two Cys residues were also very close, indicating their binding to the zinc ion. This calculation ensured that introducing a tetrahedral $Zn(Cys)_2(His)_2$ core in the topology file would not bias the structure. It also allowed determining which His N was bound to zinc. In house modifications were thus incorporated in the topology and parameter files (topallhdg.pro and parallhdg.pro) to account for for zinc binding to cysteines and histidines in a tetrahedral geometry with 2.33 Å and 2.00 Zn-S and Zn-N bonds, respectively, 109.4°, 109.4°, 105° and 125° S-Zn-S and S-Zn-N, Zn-S-C and Zn-N-C angles, respectively. Structures selected for analysis had no NOE violations greater than 0.2 Å and no dihedral angle violations greater than 5°.

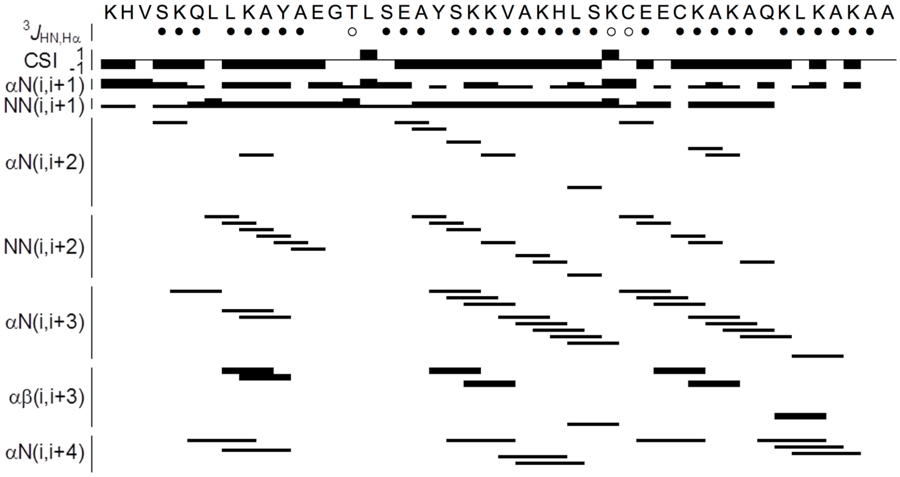

Fig. S4 Summary of ^{1}H NMR data for Zn·Lasd(HHCC) in H_2O/D_2O 9:1 (pH 6.4) at 298 K.

Table S2 ^{1}H NMR (500 MHz) coupling constants ($^3J_{HN,H\alpha}$ / Hz) and chemical shifts (δ / ppm) for Zn·Lasd(HHCC) in H_2O/D_2O 9:1 (pH 6.4) at 298 K.[a]

Residue	$^3J_{HN,H\alpha}$	δ(HN)	δ(Hα)	δ(Hβ)	δ(others)
Ac	–	–	2.018		
LYS 1	n.d.	8.040	4.100	1.576, 1.649	CH_2(γ): 1.340; CH_2(δ): 1.357; CH_2(ε): 3.008
HIS 2	n.d.	7.830	4.300	2.797, 2.902	
VAL 3	7.0	6.930	3.960	1.983	CH_3(γ): 1.065, 1.148
SER 4	< 4	8.172	4.353	4.095	
LYS 5	4.2	8.867	3.936	1.871, 1.972	CH_2(γ): 1.521; CH_2(δ): 1.752; CH_2(ε): 3.024
GLN 6	4.0	8.556	4.032	2.004, 2.111	CH_2(γ): 2.477; NH(ε): 6.908, 7.552
LEU 7	7.9	7.623	4.200	1.294, 1.908	CH_2(γ): 1.562; CH_3(δ): 0.736, 0.898
LEU 8	< 5	8.484	4.026	1.412, 1.875	CH_2(γ): 1.736; CH_3(δ): 0.873, 0.942

LYS 9	4.1	8.109	3.967	1.880, 1.937	CH_2(γ): 1.358, 1.481; CH_2(δ): 1.700; CH_2(ε): 3.036
ALA 10	< 4	7.528	4.194	1.445	
TYR 11	2.5	8.791	4.175	3.192, 3.387	CH(δ): 6.989; CH(ε): 6.866
ALA 12	3.7	8.608	3.893	1.574	
GLU 13	7.9	8.190	4.183	2.088, 2.157	CH_2(γ): 2.328, 2.599
GLY 14	–	7.819	3.979, 4.100		
THR 15	9.7	8.264	4.440	4.541	CH_3(γ): 1.117
LEU 16	7.2	7.417	4.545	1.752	CH_2(γ): 1.747; CH_3(δ): 0.965, 1.085
SER 17	3.7	8.598	4.486	4.167, 4.368	
GLU 18	2.7	9.130	4.157	2.094, 2.183	CH_2(γ): 2.302, 2.416
ALA 19	2.8	8.572	4.106	1.350	
TYR 20	7.4	7.696	4.423	3.089	CH(δ): 7.211; CH(ε): 6.899
SER 21	3.1	9.094	4.229	4.144	
LYS 22	3.7	8.169	4.155	1.953	CH_2(γ): 1.519; CH_2(δ): 1.736; CH_2(ε): 3.025
LYS 23	5.4	7.335	4.064	1.983	CH_2(γ): 1.424; CH_2(δ): 1.624; CH_2(ε): 2.896
VAL 24	4.3	8.278	3.480	1.992	CH_3(γ): 0.355, 1.040
ALA 25	2.5	8.666	3.776	1.639	
LYS 26	5.0	7.866	4.103	1.978	CH_2(γ): 1.544; CH_2(δ): 1.725; CH_2(ε): 3.009
HIS 27	5.9	7.675	4.321	3.230, 3.284	CH(δ), 6.893; CH(ε): 7.833
LEU 28	3.1	8.526	3.854	1.396, 1.799	CH_2(γ): 1.767; CH_3(δ): 0.636
SER 29	< 5	7.612	4.198	4.029	
LYS 30	9.9	7.073	4.628	1.711, 1.950	CH_2(γ): 1.362; CH_2(δ): 1.610; CH_2(ε): 2.860
CYS 31	9.1	7.525	4.643	1.858, 2.519	
GLU 32	3.7	8.993	4.087	2.075, 2.165	CH_2(γ): 2.385, 2.453
GLU 33	n.d.	8.381	4.191	1.936	CH_2(γ): 2.242
CYS 34	5.6	9.284	4.117	2.868, 3.135	
LYS 35	< 6	8.378	3.867	1.926, 2.231	CH_2(γ): 1.569; CH_2(δ): 1.412; CH_2(ε): 2.949
ALA 36	4.1	8.140	4.249	1.575	
LYS 37	< 6	7.608	4.010	1.872, 2.025	CH_2(γ): 1.434; CH_2(δ): 1.745; CH_2(ε): 2.928
ALA 38	< 6	8.487	4.047	1.649	
GLN 39	n.d.	8.240	4.071	2.198, 2.302	CH_2(γ): 2.508, 2.636; NH(ε): 6.931, 7.467
LYS 40	5.3	7.769	4.174	1.991	CH_2(γ): 1.523; CH_2(δ): 1.690; CH_2(ε): 3.015
LEU 41	< 6	7.849	4.169	1.493	CH_2(γ): 1.888; CH_3(δ): 0.862, 0.911
LYS 42	5.8	7.961	4.249	1.909	CH_2(γ): 1.572; CH_2(δ): 1.728; CH_2(ε): 3.033
ALA 43	5.6	7.998	4.310	1.472	
LYS 44	< 6	8.025	4.085	1.915, 1.990	CH_2(γ): 1.591; CH_2(δ): 1.738; CH_2(ε): 3.007
ALA 45	5.0	7.935	4.276	1.509	
ALA 46	> 6	8.045	4.284	1.469	

[a] Chemical shifts are measured relative to external DSS.

Table S3 NOE-derived distances (Å) used for calculations with X-PLOR for Zn·L_{ASD}(HHCC).

LYS	1	HN	LYS	1	HA	3.35	LYS	22	HN	LYS	22	HD*	2.90
LYS	1	HN	HIS	2	HN	3.48	LYS	22	HN	LYS	23	HN	2.74
HIS	2	HD2	LYS	1	HA	3.99	LYS	23	HB*	TYR	20	HA	2.98
HIS	2	HD2	LYS	1	HB*	4.24	LYS	23	HN	TYR	20	HA	3.13
HIS	2	HN	LYS	1	HA	2.31	LYS	23	HN	SER	21	HA	4.03
HIS	2	HB*	HIS	2	HA	2.73	LYS	23	HN	LYS	22	HA	3.27
HIS	2	HD2	HIS	2	HA	3.91	LYS	23	HN	LYS	22	HB*	3.41
HIS	2	HD2	HIS	2	HB*	2.86	LYS	23	HN	LYS	23	HA	2.74
HIS	2	HE1	HIS	2	HN	3.37	LYS	23	HN	LYS	23	HB*	2.57
HIS	2	HN	HIS	2	HA	3.70	LYS	23	HN	LYS	23	HD*	3.67
HIS	2	HN	HIS	2	HB*	2.78	LYS	23	HN	LYS	23	HG*	4.02
HIS	2	HN	HIS	2	HD2	3.39	LYS	23	HN	VAL	24	HG*	5.14
HIS	2	HN	VAL	3	HN	3.57	VAL	24	HG*	VAL	3	HG*	5.42
VAL	3	HG*	HIS	2	HA	4.72	VAL	24	HG*	LEU	8	HA	4.53
VAL	3	HG*	HIS	2	HB*	4.97	VAL	24	HG*	LEU	8	HD*	4.89
VAL	3	HN	HIS	2	HA	2.48	VAL	24	HG*	TYR	11	HA	4.18

VAL	3	HN	HIS	2	HB*	2.63
VAL	3	HG*	VAL	3	HA	3.30
VAL	3	HN	VAL	3	HA	2.94
VAL	3	HN	VAL	3	HB	2.60
VAL	3	HN	VAL	3	HG*	3.40
VAL	3	HG*	LEU	7	HB*	4.05
VAL	3	HG*	LEU	8	HA	4.64
VAL	3	HG*	LEU	8	HG	4.09
VAL	3	HG*	VAL	24	HA	3.67
VAL	3	HG*	HIS	27	HB*	4.14
SER	4	HN	VAL	3	HA	2.23
SER	4	HN	VAL	3	HG*	3.67
SER	4	HN	SER	4	HA	2.63
SER	4	HN	SER	4	HB*	2.50
SER	4	HN	LEU	7	HD*	5.79
SER	4	HN	LEU	7	HN	3.54
SER	4	HN	LEU	8	HD*	4.27
LYS	5	HN	SER	4	HA	2.51
LYS	5	HN	SER	4	HB*	3.88
LYS	5	HN	SER	4	HN	4.28
LYS	5	HN	LYS	5	HA	3.07
LYS	5	HN	LYS	5	HB*	3.03
LYS	5	HN	LYS	5	HG*	4.03
LYS	5	HN	GLN	6	HN	3.39
GLN	6	HN	SER	4	HA	3.58
GLN	6	HN	LYS	5	HA	3.45
GLN	6	HN	LYS	5	HB*	2.93
GLN	6	HN	GLN	6	HA	2.77
GLN	6	HN	GLN	6	HB*	2.84
GLN	6	HN	GLN	6	HG*	3.71
GLN	6	HN	LEU	7	HN	2.77
LEU	7	HN	VAL	3	HG*	4.96
LEU	7	HD*	SER	4	HA	4.65
LEU	7	HN	GLN	6	HB*	2.95
LEU	7	HB*	LEU	7	HA	2.81
LEU	7	HD*	LEU	7	HA	3.45
LEU	7	HN	LEU	7	HA	3.08
LEU	7	HN	LEU	7	HB*	2.66
LEU	7	HN	LEU	7	HD*	4.21
LEU	7	HN	LEU	7	HG	3.31
LEU	7	HD*	TYR	20	HB*	5.56
LEU	8	HN	VAL	3	HG*	4.13
LEU	8	HD*	LYS	5	HA	3.92
LEU	8	HN	LYS	5	HA	3.07
LEU	8	HN	LEU	7	HA	3.26
LEU	8	HN	LEU	7	HB*	3.60
LEU	8	HN	LEU	7	HG	3.34
LEU	8	HN	LEU	7	HN	2.42
LEU	8	HD*	LEU	8	HA	3.09
LEU	8	HN	LEU	8	HB*	2.69
LEU	8	HN	LEU	8	HD*	3.63
LEU	8	HN	LEU	8	HG	2.55
LEU	8	HN	LYS	9	HN	2.65
LEU	8	HN	ALA	10	HN	3.74
LEU	8	HD*	CYS	34	HB*	4.59
LYS	9	HN	LEU	7	HN	3.40
LYS	9	HN	LEU	8	HA	2.93
LYS	9	HN	LEU	8	HD*	5.09
LYS	9	HN	LYS	9	HA	2.64
LYS	9	HN	LYS	9	HB*	2.37

VAL	24	HG*	TYR	11	HB*	3.71
VAL	24	HG*	LEU	16	HB*	4.24
VAL	24	HG*	TYR	20	HA	5.37
VAL	24	HG*	SER	21	HA	4.15
VAL	24	HN	SER	21	HA	3.14
VAL	24	HN	LYS	23	HA	2.80
VAL	24	HN	LYS	23	HN	2.61
VAL	24	HB	VAL	24	HA	2.83
VAL	24	HG*	VAL	24	HA	3.06
VAL	24	HN	VAL	24	HA	2.82
VAL	24	HN	VAL	24	HB	2.22
VAL	24	HN	VAL	24	HG*	3.23
VAL	24	HG*	ALA	25	HA	4.70
VAL	24	HG*	ALA	25	HB*	4.69
VAL	24	HN	ALA	25	HB*	3.68
VAL	24	HG*	LEU	28	HD*	4.04
ALA	25	HN	TYR	11	HD*	4.48
ALA	25	HN	TYR	11	HE*	3.90
ALA	25	HN	SER	21	HA	3.70
ALA	25	HB*	LYS	22	HA	3.01
ALA	25	HN	LYS	22	HA	3.16
ALA	25	HN	LYS	23	HA	4.16
ALA	25	HN	LYS	23	HN	3.89
ALA	25	HN	VAL	24	HA	3.39
ALA	25	HN	VAL	24	HB	2.56
ALA	25	HN	VAL	24	HG*	3.92
ALA	25	HN	VAL	24	HN	2.69
ALA	25	HN	ALA	25	HA	2.79
ALA	25	HN	ALA	25	HB*	2.87
ALA	25	HN	LYS	26	HN	2.79
ALA	25	HN	HIS	27	HN	4.13
ALA	25	HN	LEU	28	HD*	5.45
LYS	26	HN	ALA	25	HA	3.37
LYS	26	HN	ALA	25	HB*	3.16
LYS	26	HN	LYS	26	HA	2.85
LYS	26	HN	LYS	26	HB*	2.43
LYS	26	HN	HIS	27	HN	2.69
HIS	27	HD2	HIS	2	HA	3.09
HIS	27	HE1	HIS	2	HA	2.72
HIS	27	HD2	VAL	3	HA	3.82
HIS	27	HD2	VAL	3	HB	3.35
HIS	27	HD2	VAL	3	HG*	3.49
HIS	27	HE1	VAL	3	HG*	6.12
HIS	27	HN	VAL	3	HG*	4.87
HIS	27	HD2	LEU	8	HD*	5.08
HIS	27	HD2	VAL	24	HA	4.31
HIS	27	HD2	VAL	24	HG*	5.62
HIS	27	HN	VAL	24	HA	3.25
HIS	27	HN	VAL	24	HG*	5.68
HIS	27	HN	LYS	26	HB*	2.93
HIS	27	HB*	HIS	27	HA	2.69
HIS	27	HD2	HIS	27	HB*	3.07
HIS	27	HN	HIS	27	HA	2.85
HIS	27	HN	HIS	27	HB*	2.59
HIS	27	HD2	LEU	28	HA	3.47
HIS	27	HD2	LEU	28	HD*	3.69
HIS	27	HD2	LEU	28	HG	3.94
HIS	27	HN	LEU	28	HD*	5.17
HIS	27	HD2	CYS	31	HB*	3.35
HIS	27	HE1	CYS	31	HB*	3.48

LYS	9	HN	ALA	10	HN	2.72
LYS	9	HN	LEU	41	HD*	4.09
ALA	10	HN	GLN	6	HA	3.33
ALA	10	HN	LYS	9	HA	3.30
ALA	10	HN	LYS	9	HB*	2.89
ALA	10	HN	ALA	10	HA	2.54
ALA	10	HN	ALA	10	HB*	2.40
ALA	10	HB*	LEU	16	HA	4.05
ALA	10	HN	LEU	16	HD*	4.88
TYR	11	HE*	VAL	3	HG*	4.92
TYR	11	HB*	LEU	8	HA	2.76
TYR	11	HD*	LEU	8	HA	3.64
TYR	11	HD*	LEU	8	HD*	5.57
TYR	11	HN	LEU	8	HA	3.15
TYR	11	HN	LYS	9	HA	3.98
TYR	11	HN	LYS	9	HN	4.00
TYR	11	HN	ALA	10	HA	3.08
TYR	11	HN	ALA	10	HB*	3.40
TYR	11	HN	ALA	10	HN	2.74
TYR	11	HB*	TYR	11	HA	2.62
TYR	11	HD*	TYR	11	HA	2.76
TYR	11	HD*	TYR	11	HB*	2.78
TYR	11	HE*	TYR	11	HB*	3.63
TYR	11	HN	TYR	11	HA	2.77
TYR	11	HN	TYR	11	HB*	2.49
TYR	11	HN	TYR	11	HD*	3.76
TYR	11	HD*	ALA	12	HA	3.56
TYR	11	HD*	ALA	12	HB*	4.44
TYR	11	HE*	ALA	12	HA	4.12
TYR	11	HE*	ALA	12	HB*	5.21
TYR	11	HN	ALA	12	HB*	4.65
TYR	11	HN	ALA	12	HN	2.73
TYR	11	HN	GLU	13	HN	4.08
TYR	11	HD*	LEU	16	HD*	3.96
TYR	11	HD*	LEU	16	HG	3.59
TYR	11	HE*	LEU	16	HD*	5.73
TYR	11	HN	LEU	16	HD*	3.99
TYR	11	HN	LEU	16	HG	3.42
TYR	11	HD*	SER	21	HA	3.02
TYR	11	HE*	SER	21	HA	3.51
TYR	11	HE*	SER	21	HB*	3.45
TYR	11	HD*	VAL	24	HB	3.22
TYR	11	HD*	VAL	24	HG*	4.06
TYR	11	HE*	VAL	24	HB	3.64
TYR	11	HE*	VAL	24	HG*	4.95
TYR	11	HN	VAL	24	HG*	4.24
TYR	11	HE*	ALA	25	HA	4.11
TYR	11	HE*	ALA	25	HB*	3.53
TYR	11	HD*	LEU	28	HD*	4.61
TYR	11	HE*	LEU	28	HD*	4.05
TYR	11	HD*	ALA	38	HB*	3.91
ALA	12	HN	LEU	8	HA	3.38
ALA	12	HB*	LYS	9	HA	3.26
ALA	12	HN	LYS	9	HA	3.21
ALA	12	HN	ALA	10	HN	4.27
ALA	12	HN	TYR	11	HB*	2.74
ALA	12	HN	TYR	11	HD*	3.50
ALA	12	HN	ALA	12	HA	2.82
ALA	12	HN	ALA	12	HB*	2.86
ALA	12	HN	GLU	13	HN	2.72
HIS	27	HD2	CYS	34	HA	3.82
HIS	27	HD2	CYS	34	HB*	2.69
LEU	28	HN	VAL	3	HG*	5.28
LEU	28	HN	VAL	24	HA	3.86
LEU	28	HN	VAL	24	HG*	5.10
LEU	28	HD*	ALA	25	HA	3.86
LEU	28	HG	ALA	25	HA	2.50
LEU	28	HN	ALA	25	HA	3.15
LEU	28	HN	LYS	26	HN	3.93
LEU	28	HN	HIS	27	HA	3.81
LEU	28	HN	HIS	27	HB*	2.99
LEU	28	HN	HIS	27	HD2	3.96
LEU	28	HN	HIS	27	HN	2.64
LEU	28	HB*	LEU	28	HA	2.84
LEU	28	HD*	LEU	28	HA	2.99
LEU	28	HD*	LEU	28	HB*	3.61
LEU	28	HN	LEU	28	HA	2.80
LEU	28	HN	LEU	28	HB*	3.25
LEU	28	HN	LEU	28	HD*	3.73
LEU	28	HN	LEU	28	HG	2.24
LEU	28	HN	SER	29	HN	2.73
LEU	28	HN	LYS	30	HN	4.22
LEU	28	HD*	CYS	31	HB*	5.58
LEU	28	HD*	CYS	34	HA	4.76
LEU	28	HD*	CYS	34	HB*	3.54
LEU	28	HD*	LYS	35	HB*	5.32
LEU	28	HD*	ALA	38	HB*	3.82
SER	29	HN	ALA	25	HA	3.73
SER	29	HN	LYS	26	HA	2.68
SER	29	HN	LEU	28	HA	3.16
SER	29	HN	LEU	28	HB*	4.02
SER	29	HN	LEU	28	HD*	4.75
SER	29	HN	SER	29	HA	2.65
SER	29	HN	SER	29	HB*	2.70
SER	29	HN	LYS	30	HN	2.64
LYS	30	HN	HIS	27	HA	3.51
LYS	30	HN	LEU	28	HA	3.53
LYS	30	HN	SER	29	HA	3.34
LYS	30	HN	SER	29	HB*	3.70
LYS	30	HB*	LYS	30	HA	2.62
LYS	30	HN	LYS	30	HB*	2.83
LYS	30	HN	LYS	30	HG*	3.25
LYS	30	HN	CYS	31	HB*	3.81
CYS	31	HB*	LEU	28	HA	3.43
CYS	31	HN	LEU	28	HA	3.03
CYS	31	HN	LYS	30	HB*	3.39
CYS	31	HN	LYS	30	HN	2.20
CYS	31	HB*	CYS	31	HA	2.63
CYS	31	HN	CYS	31	HB*	2.61
CYS	31	HB*	CYS	34	HB*	3.12
GLU	32	HN	CYS	31	HA	2.24
GLU	32	HN	CYS	31	HB*	3.98
GLU	32	HN	CYS	31	HN	3.83
GLU	32	HN	GLU	32	HA	2.83
GLU	32	HN	GLU	32	HB*	2.71
GLU	32	HN	GLU	32	HG*	3.21
GLU	32	HN	GLU	33	HN	2.74
GLU	33	HN	CYS	31	HA	3.22
GLU	33	HN	CYS	31	HN	4.25
GLU	33	HN	GLU	32	HB*	2.56

ALA	12	HN	GLY	14	HN	3.85
ALA	12	HN	VAL	24	HG*	5.41
ALA	12	HN	LEU	41	HD*	5.22
GLU	13	HN	ALA	12	HA	3.36
GLU	13	HN	ALA	12	HB*	3.01
GLU	13	HG*	GLU	13	HA	3.46
GLU	13	HN	GLU	13	HA	2.74
GLU	13	HN	GLU	13	HB*	2.60
GLU	13	HN	GLU	13	HG*	2.79
GLU	13	HN	GLY	14	HN	2.54
GLY	14	HN	GLU	13	HA	2.79
GLY	14	HN	GLY	14	HA*	2.53
THR	15	HG*	ALA	10	HA	4.48
THR	15	HN	ALA	10	HA	2.81
THR	15	HN	ALA	10	HB*	3.71
THR	15	HG2*	GLU	13	HB*	3.73
THR	15	HN	GLY	14	HA1	2.98
THR	15	HN	GLY	14	HN	2.55
THR	15	HG2*	THR	15	HA	3.04
THR	15	HN	THR	15	HA	2.81
THR	15	HN	THR	15	HB	3.37
THR	15	HN	THR	15	HG2*	3.28
THR	15	HN	LEU	16	HG	2.93
THR	15	HN	LEU	16	HN	2.46
LEU	16	HD*	ALA	10	HB*	4.00
LEU	16	HN	ALA	10	HB*	4.20
LEU	16	HD*	TYR	11	HA	3.10
LEU	16	HD*	TYR	11	HB*	3.98
LEU	16	HN	TYR	11	HA	3.21
LEU	16	HN	THR	15	HA	3.11
LEU	16	HN	THR	15	HG2*	4.51
LEU	16	HB*	LEU	16	HA	2.60
LEU	16	HD*	LEU	16	HA	3.67
LEU	16	HN	LEU	16	HA	2.80
LEU	16	HN	LEU	16	HD*	4.05
LEU	16	HD*	TYR	20	HA	4.33
LEU	16	HD*	TYR	20	HB*	3.47
LEU	16	HD*	SER	21	HA	3.70
SER	17	HN	ALA	10	HB*	4.57
SER	17	HN	LEU	16	HA	2.29
SER	17	HN	LEU	16	HB*	2.91
SER	17	HN	LEU	16	HD*	3.75
SER	17	HN	LEU	16	HN	3.84
SER	17	HN	SER	17	HA	2.89
SER	17	HN	SER	17	HB*	2.79
SER	17	HN	TYR	20	HB*	3.48
SER	17	HN	TYR	20	HD*	4.85
SER	17	HN	TYR	20	HN	3.88
GLU	18	HN	SER	17	HA	2.66
GLU	18	HN	SER	17	HB*	3.46
GLU	18	HN	SER	17	HN	4.53
GLU	18	HN	GLU	18	HA	2.75
GLU	18	HN	GLU	18	HB*	2.94
GLU	18	HN	GLU	18	HG*	3.99
GLU	18	HN	ALA	19	HB*	5.19
GLU	18	HN	ALA	19	HN	3.64
ALA	19	HN	GLU	18	HA	3.24
ALA	19	HN	GLU	18	HB*	3.19
ALA	19	HN	ALA	19	HA	2.82
ALA	19	HN	ALA	19	HB*	3.02
GLU	33	HN	GLU	33	HA	2.65
GLU	33	HN	GLU	33	HB*	3.21
CYS	34	HN	LEU	8	HD*	6.05
CYS	34	HN	HIS	27	HD2	3.93
CYS	34	HB*	LEU	28	HA	3.53
CYS	34	HN	LEU	28	HD*	5.03
CYS	34	HN	CYS	31	HA	3.94
CYS	34	HN	CYS	31	HB*	3.47
CYS	34	HN	GLU	32	HN	4.15
CYS	34	HN	GLU	33	HA	3.42
CYS	34	HN	GLU	33	HB*	3.14
CYS	34	HN	GLU	33	HG*	4.09
CYS	34	HN	GLU	33	HN	2.36
CYS	34	HB*	CYS	34	HA	2.67
CYS	34	HN	CYS	34	HA	2.77
CYS	34	HN	CYS	34	HB*	2.65
CYS	34	HN	LYS	35	HA	3.88
CYS	34	HN	ALA	36	HN	4.02
CYS	34	HN	LYS	37	HN	4.25
LYS	35	HN	LEU	28	HD*	4.33
LYS	35	HN	GLU	32	HA	2.69
LYS	35	HN	CYS	34	HB*	3.08
LYS	35	HB*	LYS	35	HA	2.91
LYS	35	HN	LYS	35	HA	2.69
LYS	35	HN	LYS	35	HB*	2.46
LYS	35	HN	LYS	35	HG*	3.80
LYS	35	HN	ALA	36	HN	2.67
LYS	35	HN	LYS	37	HN	3.77
ALA	36	HN	GLU	32	HA	3.15
ALA	36	HB*	GLU	33	HA	2.89
ALA	36	HN	GLU	33	HA	3.77
ALA	36	HN	LYS	35	HA	3.18
ALA	36	HN	LYS	35	HB*	2.73
ALA	36	HN	ALA	36	HA	2.87
ALA	36	HN	ALA	36	HB*	2.79
ALA	36	HN	LYS	37	HN	2.73
LYS	37	HN	LEU	8	HD*	4.84
LYS	37	HN	ALA	36	HA	3.12
LYS	37	HN	ALA	36	HB*	3.34
LYS	37	HN	LYS	37	HB*	2.92
LYS	37	HN	LYS	37	HG*	2.91
LYS	37	HN	ALA	38	HB*	4.17
ALA	38	HN	LEU	28	HD*	4.51
ALA	38	HB*	LYS	35	HA	3.24
ALA	38	HN	LYS	35	HA	3.19
ALA	38	HN	LYS	37	HB*	2.83
ALA	38	HN	LYS	37	HB*	2.92
ALA	38	HN	LYS	37	HN	3.01
ALA	38	HN	ALA	38	HA	2.41
ALA	38	HN	ALA	38	HB*	2.89
ALA	38	HN	GLN	39	HN	2.67
ALA	38	HN	LYS	40	HN	3.86
GLN	39	HN	ALA	36	HA	2.72
GLN	39	HN	ALA	36	HB*	4.05
GLN	39	HN	ALA	38	HB*	3.26
GLN	39	HN	GLN	39	HA	2.59
GLN	39	HN	GLN	39	HB*	2.66
GLN	39	HN	GLN	39	HB*	3.11
GLN	39	HN	LYS	40	HN	2.70
LYS	40	HN	LYS	37	HA	2.95

ALA	19	HB*	TYR	20	HA	4.55	LYS	40	HN	GLN	39	HA	3.20
ALA	19	HN	TYR	20	HN	2.92	LYS	40	HN	GLN	39	HB*	3.05
TYR	20	HD*	LEU	7	HD*	4.96	LYS	40	HN	LYS	40	HA	2.67
TYR	20	HE*	LEU	7	HD*	5.26	LYS	40	HN	LYS	40	HB*	2.64
TYR	20	HB*	LEU	16	HA	3.92	LEU	41	HD*	LEU	8	HB*	3.72
TYR	20	HD*	LEU	16	HD*	5.00	LEU	41	HD*	LYS	9	HA	3.59
TYR	20	HN	LEU	16	HD*	5.18	LEU	41	HD*	LYS	9	HE*	5.15
TYR	20	HN	GLU	18	HA	3.41	LEU	41	HD*	ALA	12	HB*	4.49
TYR	20	HD*	ALA	19	HB*	5.02	LEU	41	HD*	ALA	38	HA	3.77
TYR	20	HN	ALA	19	HB*	3.36	LEU	41	HN	ALA	38	HA	2.78
TYR	20	HB*	TYR	20	HA	2.68	LEU	41	HD*	LEU	41	HA	3.61
TYR	20	HD*	TYR	20	HA	3.90	LEU	41	HD*	LEU	41	HB*	4.00
TYR	20	HD*	TYR	20	HB*	4.34	LEU	41	HN	LEU	41	HA	2.66
TYR	20	HN	TYR	20	HA	2.85	LEU	41	HN	LEU	41	HB*	3.05
TYR	20	HN	TYR	20	HB*	2.70	LEU	41	HN	LEU	41	HD*	3.55
TYR	20	HN	TYR	20	HD*	4.25	LEU	41	HN	LEU	41	HG	2.29
TYR	20	HD*	VAL	24	HG*	5.68	LYS	42	HN	LEU	41	HA	4.31
SER	21	HN	TYR	11	HD*	5.06	LYS	42	HN	LYS	42	HA	2.77
SER	21	HN	LEU	16	HD*	4.48	LYS	42	HN	ALA	45	HB*	4.18
SER	21	HN	LEU	16	HN	4.85	ALA	43	HN	GLN	39	HA	3.55
SER	21	HN	SER	17	HN	4.43	ALA	43	HB*	LYS	40	HA	2.79
SER	21	HN	ALA	19	HA	3.73	ALA	43	HN	LYS	42	HA	2.98
SER	21	HN	ALA	19	HB*	4.87	ALA	43	HN	LYS	42	HB*	3.20
SER	21	HN	ALA	19	HN	4.21	ALA	43	HN	ALA	43	HA	3.08
SER	21	HN	TYR	20	HA	3.35	ALA	43	HN	ALA	43	HB*	3.44
SER	21	HN	TYR	20	HB*	3.19	LYS	44	HN	LYS	40	HA	3.33
SER	21	HN	TYR	20	HD*	5.66	LYS	44	HN	LEU	41	HA	3.33
SER	21	HN	TYR	20	HN	2.54	LYS	44	HN	LEU	41	HD*	5.00
SER	21	HN	SER	21	HA	2.82	LYS	44	HN	LYS	44	HA	2.52
SER	21	HN	SER	21	HB*	2.74	LYS	44	HN	LYS	44	HB*	2.53
SER	21	HN	LYS	22	HN	2.84	ALA	45	HB*	LEU	41	HA	2.86
SER	21	HN	LYS	23	HN	4.00	ALA	45	HN	LEU	41	HA	2.95
SER	21	HN	VAL	24	HG*	6.08	ALA	45	HN	LYS	44	HA	2.69
SER	21	HN	VAL	24	HN	4.62	ALA	45	HN	LYS	44	HB*	3.31
LYS	22	HN	TYR	20	HN	3.80	ALA	45	HN	ALA	45	HA	2.87
LYS	22	HN	LYS	22	HA	2.59	ALA	45	HN	ALA	45	HB*	2.87
LYS	22	HN	LYS	22	HB*	2.63	ALA	46	HN	ALA	46	HB*	3.65

Table S4 Dihedral angle restraints (°) used in calculations with X-PLOR for Zn·L_{ASD}(HHCC).

VAL	3	C	SER	4	N	SER	4	CA	SER	4	C	-65	-90	-40
SER	4	C	LYS	5	N	LYS	5	CA	LYS	5	C	-65	-90	-40
LYS	5	C	GLN	6	N	GLN	6	CA	GLN	6	C	-65	-90	-40
LEU	7	C	LEU	8	N	LEU	8	CA	LEU	8	C	-65	-90	-40
SER	8	C	LYS	9	N	LYS	9	CA	LYS	9	C	-65	-90	-40
LYS	9	C	ALA	10	N	ALA	10	CA	ALA	10	C	-65	-90	-40
ALA	10	C	TYR	11	N	TYR	11	CA	TYR	11	C	-65	-90	-40
TYR	11	C	ALA	12	N	ALA	12	CA	ALA	12	C	-65	-90	-40
GLY	14	C	THR	15	N	THR	15	CA	THR	15	C	-120	-160	-80
LEU	16	C	SER	17	N	SER	17	CA	SER	17	C	-65	-90	-40
SER	17	C	GLU	18	N	GLU	18	CA	GLU	18	C	-65	-90	-40
GLU	18	C	ALA	19	N	ALA	19	CA	ALA	19	C	-65	-90	-40
TYR	20	C	SER	21	N	SER	21	CA	SER	21	C	-65	-90	-40
SER	21	C	LYS	22	N	LYS	22	CA	LYS	22	C	-65	-90	-40
LYS	22	C	LYS	23	N	LYS	23	CA	LYS	23	C	-65	-90	-40
LYS	23	C	VAL	24	N	VAL	24	CA	VAL	24	C	-65	-90	-40
VAL	24	C	ALA	25	N	ALA	25	CA	ALA	25	C	-65	-90	-40
ALA	25	C	LYS	26	N	LYS	26	CA	LYS	26	C	-65	-90	-40
LYS	26	C	HIS	27	N	HIS	27	CA	HIS	27	C	-65	-90	-40
HIS	27	C	LEU	28	N	LEU	28	CA	LEU	28	C	-65	-90	-40

LEU	28	C	SER	29	N	SER	29	CA	SER	29	C	-65	-90	-40
SER	29	C	LYS	30	N	LYS	30	CA	LYS	30	C	-120	-160	-80
LYS	30	C	CYS	31	N	CYS	31	CA	CYS	31	C	-120	-160	-80
CYS	31	C	GLU	32	N	GLU	32	CA	GLU	32	C	-65	-90	-40
GLU	33	C	CYS	34	N	CYS	34	CA	CYS	34	C	-65	-90	-40
CYS	34	C	LYS	35	N	LYS	35	CA	LYS	35	C	-65	-90	-40
LYS	35	C	ALA	36	N	ALA	36	CA	ALA	36	C	-65	-90	-40
ALA	36	C	LYS	37	N	LYS	37	CA	LYS	37	C	-65	-90	-40
LYS	37	C	ALA	38	N	ALA	38	CA	ALA	38	C	-65	-90	-40
GLN	39	C	LYS	40	N	LYS	40	CA	LYS	40	C	-65	-90	-40
LYS	40	C	LEU	41	N	LEU	41	CA	LEU	41	C	-65	-90	-40
LEU	41	C	LYS	42	N	LYS	42	CA	LYS	42	C	-65	-90	-40
LYS	42	C	ALA	43	N	ALA	43	CA	ALA	43	C	-65	-90	-40
ALA	43	C	LYS	44	N	LYS	44	CA	LYS	44	C	-65	-90	-40
LYS	44	C	ALA	45	N	ALA	45	CA	ALA	45	C	-65	-90	-40

References

[1] G. Schwarzenbach, H. Flaschka, *Complexometric Titrations*, Methuen, London, **1969**.
[2] P. W. Riddles, R. L. Blakeley, B. Zerner, *Methods Enzymol.* **1983**, *91*, 49–60.
[3] E. Bourlès, M. Isaac, C. Lebrun, J.-M. Latour, O. Sénèque, *Chem.-Eur. J.* **2011**, *17*, 13762–13772.
[4] K. Mikkelsen, S. Nielsen, *J. Phys. Chem.* **1960**, *64*, 632–637.
[5] V. Lebrun, A. Tron, L. Scarpantonio, C. Lebrun, J.-L. Ravanat, J.-M. Latour, N. D. McClenaghan, O. Sénèque, *Angew. Chem., Int. Ed.* **2014**, *53*, 9365–9368.
[6] V. Lebrun, A. Tron, C. Lebrun, J.-M. Latour, N. D. McClenaghan, O. Sénèque, *Chem.-Eur. J.* **2015**, *21*, 14002–14010.
[7] V. Sklenar, M. Piotto, R. Leppik, V. Saudek, *J. Magn. Reson. Ser. A* **1993**, *102*, 241–245.
[8] M. Piotto, V. Saudek, V. Sklenar, *J. Biomol. NMR* **1992**, *2*, 661–665.
[9] T. D. Goddard, D. G. Kneller, *SPARKY 3.1*, University of California, San Francisco, **n.d.**
[10] A. T. Brünger, *A System for X-Ray Crystallography and NMR. X-PLOR, Version 3.1*, Yale University Press, New Haven, **1992**.
